# Actual European forest management by region, tree species and owner based on 714,000 re-measured trees in national forest inventories

**DOI:** 10.1371/journal.pone.0207151

**Published:** 2018-11-12

**Authors:** Mart-Jan Schelhaas, Jonas Fridman, Geerten M. Hengeveld, Helena M. Henttonen, Aleksi Lehtonen, Uwe Kies, Nike Krajnc, Bas Lerink, Áine Ní Dhubháin, Heino Polley, Thomas A. M. Pugh, John J. Redmond, Brigitte Rohner, Cristian Temperli, Jordi Vayreda, Gert-Jan Nabuurs

**Affiliations:** 1 Wageningen Environmental Research (WENR, previously Alterra), Wageningen University and Research, Wageningen, The Netherlands; 2 Swedish University of Agricultural Sciences (SLU), Umeå, Sweden; 3 Biometris, Wageningen University and Research, Wageningen, The Netherlands; 4 Forest and Nature Conservation Policy Group Wageningen University and Research, Wageningen, The Netherlands; 5 Natural Resources Institute Finland (Luke), Helsinki, Finland; 6 InnovaWood, Brussels, Belgium; 7 Slovenian Forestry Institute, Ljubljana, Slovenia; 8 Forest Ecology and Forest Management Group, Wageningen University and Research, Wageningen, The Netherlands; 9 Forestry Section, School of Agriculture and Food Science, University College Dublin, Belfield, Dublin, Ireland; 10 Thünen Institute of Forest Ecosystems, Eberswalde, Germany; 11 School of Geography, Earth & Environmental Sciences, University of Birmingham, Birmingham, United Kingdom; 12 Birmingham Institute of Forest Research, University of Birmingham, Birmingham, United Kingdom; 13 Department of Agriculture, Food and the Marine, Wexford, Ireland; 14 Resource Analysis, Swiss Federal Institute for Forest, Snow and Landscape Research (WSL) Birmensdorf, Switzerland; 15 CREAF, Cerdanyola del Valles, Spain; Albert-Ludwigs-Universitat Freiburg, GERMANY

## Abstract

**Background:**

European forests have a long record of management. However, the diversity of the current forest management across nations, tree species and owners, is hardly understood. Often when trying to simulate future forest resources under alternative futures, simply the yield table style of harvesting is applied. It is now crucially important to come to grips with actual forest management, now that demand for wood is increasing and the EU Land Use, Land Use Change and Forestry Regulation has been adopted requiring ‘continuation of current management practices’ as a baseline to set the Forest Reference Level carbon sink.

**Methods:**

Based on a large dataset of 714,000 re-measured trees in National Forest inventories from 13 regions, we are now able to analyse actual forest harvesting.

**Conclusions:**

From this large set of repeated tree measurements we can conclude that there is no such thing as yield table harvesting in Europe. We found general trends of increasing harvest probability with higher productivity of the region and the species, but with important deviations related to local conditions like site accessibility, state of the forest resource (like age), specific subsidies, importance of other forest services, and ownership of the forest. As a result, we find a huge diversity in harvest regimes. Over the time period covered in our inventories, the average harvest probability over all regions was 2.4% yr-1 (in number of trees) and the mortality probability was 0.4% yr-1. Our study provides underlying and most actual data that can serve as a basis for quantifying ‘continuation of current forest management’. It can be used as a cornerstone for the base period as required for the Forest Reference Level for EU Member States.

## Introduction

European forests have a long record of management [1–3]. The earliest evidence dates some 7000 years back to prehistoric man who already had an influence on the early Holocene forests. After millennia of degradation and deforestation, wood shortages were apparent as early as in the Roman era and in early Medieval times. The unregulated felling and grazing that were the basis for wood shortages led to the introduction of controls. For example, a code in early Irish Law (approximately the 8th century A.D.) set out penalties for the felling or damaging of privately owned trees, listing eight classes of trees of varying `nobility' [4]. This is probably the first time a written ‘management regime’ was published. In Switzerland the oldest records of protection forests, where all cutting was prohibited to prevent avalanches and landslides, go back to 1339 [5]. It was much later, in 1713, after long periods of further degradation in Medieval times, that Hans Carl von Carlowitz with his book “Sylvicultura oeconomica” laid a basis for a scientific approach to sustainable management leading to the development of formal forestry education. The fear of over-exploitation of forests was also a major driving force for the development of forest inventories. The earliest attempts at the scale of forest estates and regions originated in Europe during the late Medieval times, whilst the first National Forest Inventories (NFIs) emerged in the early twentieth century in northern Europe [6].

Today, sustainable forest management in Europe, in all its variety, is planned and monitored, and aims at achieving a variety of functions from wood production and recreation to carbon sequestration and nature conservation, at varying spatial and temporal scales. Furthermore, the management decisions influence growth, forest composition and structure, and produced commodities, whilst also affecting individual tree mortality rates and the risk of large-scale disturbances (fire, storm, snow, pests) [7].

In order to support the planning by forest managers, yield tables were developed that were based on long term monitoring plots [8,9], the earliest dating back to the early 19^th^ century [10]. Each country developed these yield tables therefore resulting in a large variety of tables, representing the variety in growth rates and management styles. Furthermore these yield tables became a basis for forest management planning at enterprise and country level. These static yield tables were very much in use in many EU countries until probably the nineties and were seen as a management guidance and a standard ‘handbook’ form of harvesting, defining the optimum point in terms of wood production. Currently their real use varies a lot between countries. Because of a lack of other information, this handbook form of harvesting is often applied in large-scale empirical forest models at country or EU level. It is then assumed that it reflects the past and/or current practices [11–13]. In other cases harvesting is only defined in terms of intensity while ignoring species- and size class-specific removal probabilities [14]. In many applications of process-based models, harvesting is also incorporated based on data from growth and yield trials [15–17], in a very simple manner or hardly at all [18,19].

The assumption that all forest harvesting is carried out according to this handbook intensity is unsupported because in practice many other factors than maximising yield play a role. The more than 16 million private forest owners and thousands of public owners in Europe each have their own management goals while decisions to harvest or not are further influenced by wood prices, state of the forest resource, available subsidies, calamities, accessibility of the site, family circumstances, etc. [20–23]. Though hugely important, at European level little is known yet about actual harvesting behaviour in the forest. Another research gap relates to natural mortality. Separating natural (incl. single-tree and large scale, disturbance-induced) and management-induced mortality is still a major challenge which we hardly understand (e.g. [24,25]). National forest inventories may hold that information, but in international statistics much of the detailed information collected in the countries is aggregated to one number (e.g. national felling/growth ratio), which means that the detailed information is lost. In the latest state of Europe’s forests, 10 countries did not even provide the net annual increment and certainly not in a harmonised way [26].

An additional reason why assessing actual harvesting behaviour is so important originates from the new Land Use, Land Use Change and Forestry (LULUCF) regulation which was adopted in May 2018 by the European Council [27]. It regulates a ‘no debit’ target for LULUCF (Forests and Agricultural soils) against a modelled future Forest Reference Level (FRL). The modelled projections for forest management and wood harvesting to set the FRL should be based on ‘*sustainable forest management practice*’ as documented in the period 2000–2009. Furthermore, Member States have to provide an accounting plan. The elements for a national forestry accounting plan should among others contain

- *‘documentary information on* ***sustainable*** *forest management* ***practices*** *and* ***intensity*** *and adopted national policies;*
- *information on how harvesting rates are expected to develop under different policy scenarios’*.

Thus, insights into real harvesting behaviour are ever more important because Member States have to quantify their Forest Reference Level under a *‘continuation of current management practices as documented in the period from 2000 to 2009’*. The additional guidance of how countries should interpret this is currently being written, but in any case basic data of real management will be valuable.

Repeated inventory data can thus help to reveal what in reality is happening in the forest and can be used to document current management practices in all its variety across species, owners and countries. We gathered a large set of repeated measurements on individual trees from permanent NFI plots, from 13 regions covering 9 European countries. It is now possible, for the first time, to do such analysis based on a large set of repeated measurements a wide range of management and growing conditions.

At these permanent NFI plots, individual trees are repeatedly measured. Harvesting will result in missing trees (i.e. stumps) in subsequent inventories. We hypothesise these missing trees and their recorded status of ‘dead’ or ‘harvested’ will reflect the pattern of harvesting and mortality within the respective stratum of the NFI. Stratification by ownership, tree species and/or diameter class then allows to infer harvesting and mortality patterns. In order to understand to what extent the actual harvesting reflects the yield table harvesting we compare these harvesting patterns to the patterns prescribed by growth and yield tables. From this comparison we can analyse harvesting by ownership and tree species in each region.

The overall aim of the study was to assess the harvest intensity (expressed as an annual probability) in European forests around the reference period (2000–2009), to compare patterns amongst regions, owners, tree species and diameter classes, and to compare patterns to “handbook” harvesting intensities. The second aim was to describe observed patterns of natural mortality in relation to harvesting patterns. We did this through: 1) developing and demonstrating an approach (see data and methods) as to how data from repeated NFI measurements can be used to derive information on the actual harvesting and natural mortality of the forest, and 2) applying the approach to a range of regions in Europe to illustrate the variety of patterns of harvesting and natural mortality at present.

## Data and methods

### Data

**Fig 1:**
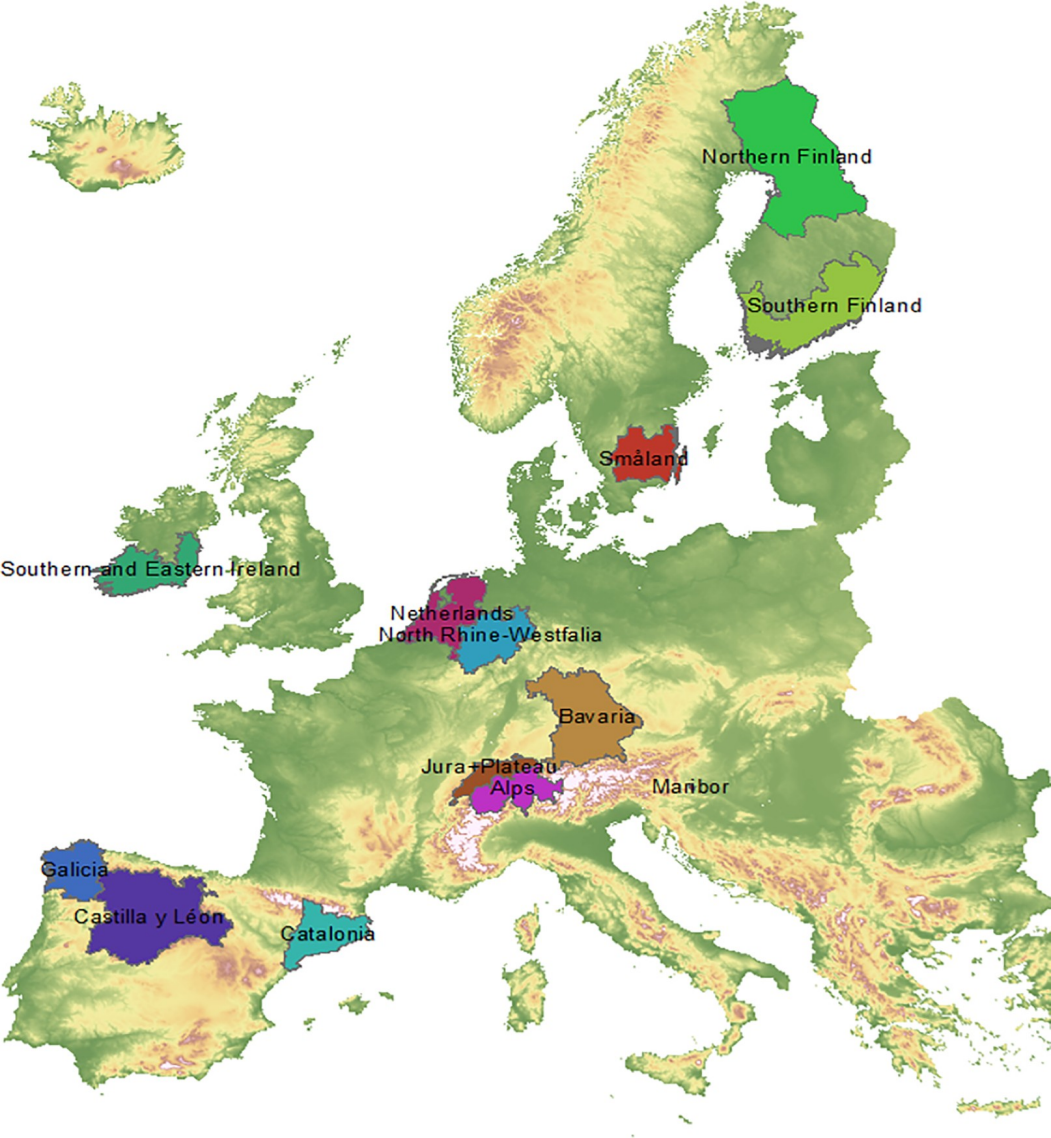
Location of the 13 regions included in this study.

From the European National Forest Inventories that we had access to, we selected 13 regions based on the following criteria: 1) Availability of repeated observations of tree status with at least the classes alive, dead and harvested recorded, 2) a preferred number of observations of at least 20,000 individual trees and 3) spanning a wide range of conditions in Europe with regard to location, climate, tree species distribution, ownership, management practices and forest history. The sampling intensity varies considerably in Europe, with generally a low number of plots per unit area in northern Europe and a high number of plots in Central Europe. As a consequence, the regions vary in size to be able to cover the minimum requirement of 20,000 trees. The total forest area per region ranges from 97,000 ha in Maribor (Slovenia) to almost 8.2 million ha in Northern Finland (Table 1, Fig 1). In total, we included more than 700,000 repeated tree observations. Northern Finland includes the production regions Southern Lapland, Kainuu and Pohjois-Pohjanmaa, while Southern Finland includes the production regions Rannikko (south), Lounais-Suomi, Häme-Uusimaa, Kaakkois-Suomi and Etelä-Savo [28]. The five production regions in Switzerland [29] were grouped in two regions to have at least 20,000 trees: the Alps region, including Prealps, Alps and southern Alps, and the region Jura+Plateau. NFIs differed in field sampling design and sample plot type. Diameter at breast height (DBH) of individual trees was always measured at 1.3 m height, but the threshold to be included differed among NFIs. Germany and Finland used an angle count method [30], while other countries used a design with circular plots, either with a variable radius depending on the plot conditions, or with different radii with corresponding diameter thresholds. More information on the Finnish NFI can be found in [28], on the Swedish NFI in [31], on the Dutch NFI in [32,33], on the German NFI in [34], on the Irish NFI in [35], on the Swiss NFI in [36], on the Spanish NFI in [37] and on the Slovenian NFI in [38].

**Table 1:** Overview data per region.

Biogeographic region [39]	Country	Region	Private ownership	Forest area (1000 ha)	NFI number	Dates of NFI measurements	mean and standard deviation of interval length (yr)	Nr of forest plots	Nr of trees	Plot radius^1^ (m)	Diameter threshold^1^ (cm)
Boreal	Finland	Northern Finland	52%	8185	NFI10/NFI11	2004-2008/ 2009–2013	4.5	3369	23225	angle count method^2^	
Boreal	Finland	Southern Finland	92%	4197	NFI10/NFI11	2004-2008/ 2009–2013	4.9	2914	22502	angle count method^2^	
Boreal	Sweden	Småland	84%	1986	NFI7-8/NFI8-9	2005-2009/ 2010–2014	5.0 (0)	2020	35094	3.5/10	4/10
Continental/Alpine	Germany	Bavaria	69%	2605	NFI2/NFI3	2000-2002/ 2011–2012	10.0 (0.67)	7895	61240	angle count method^2^	7
Continental/Alpine	Switzerland	Alps	31%	799	NFI2/NFI3	1993-1996/ 2004–2006	11.3 (1.14)	3169	36768	8/12.6	12/36
Continental/Alpine	Switzerland	Jura+Plateau	34%	458	NFI2/NFI3	1993-1996/ 2004–2006	10.3 (0.67)	2034	24235	8/12.6	12/36
Continental/Alpine	Slovenia	Maribor	54%	97	NFI1/NFI2	1992–2002 / 2002–2012	10	8036	108715	8/12.6	10/30
Atlantic	Netherlands	Netherlands	51%	373	NFI5/NFI6	2001-2005/ 2012–2013	9.6 (1.56)	1217	25562	variable (5–20 m)	5
Atlantic	Germany	North Rhine-Westphalia	83%	608	NFI2/NFI3	2000-2002/ 2011–2012	10.2 (0.60)	2287	14383	angle count method^2^	7
Atlantic	Ireland	Southern and Eastern Ireland	39%	324	NFI1/NFI2	2004-2006/ 2009–2012	6.0 (0.91)	580	11395	3/7/12.62	7/12/20
Atlantic	Spain	Galicia	99%	1396	NFI2/NFI3	1986-1987/ 1997–1998	11.0 (0.02)	3941	75929	5/10/15/25	7.5/12.5/22.5/42.5
Mediterranean	Spain	Catalonia	81%	1541	NFI2/NFI3	1989-1990/ 2000–2001	11.3 (0.68)	8471	138831	5/10/15/25	7.5/12.5/22.5/42.5
Mediterranean	Spain	Castilla y León	47%	2435	NFI2/NFI3	1991-1992/ 2002–2004	11.5 (1.13)	9224	136773	5/10/15/25	7.5/12.5/22.5/42.5
Total				24923			7.2	53796	714652		

1 Multiple diameter thresholds indicate a design with plots consisting of concentric circles with their radii and the corresponding thresholds

2 see for details on this method for example [30].

## Methods

### Data preparation

All tree observations were merged into one database, except for the Finnish data which was handled separately for data protection reasons. Each record contained information on the region, plot ID, tree number, original species name, DBH at first measurement (in mm), original status at the second observation and original owner class. For each record we added how many trees were represented by this observation using the following equation:

$$M_{ij}=N_{ij}\times A_{j}/P_{j},$$ (1)

where *M*_*ij*_ is the total number of trees in region *j* represented by observation *i*, *N*_*ij*_ the number of trees per ha represented by this observation, *A*_*j*_ the total forest area and *P*_*j*_ the total number of plots in region *j* (Table 1). *N*_*ij*_ was given in the original NFI data in case of the angle count method, and for the other cases derived from the plot area, using the appropriate plot radius given the DBH of the tree (Table 1). Records that were incomplete or where the status of the tree (live/dead/harvested) could not be determined were deleted from the database.

Tree records were harmonised for the names of the species. First, scientific names were attached to each tree record if not available yet, and existing scientific names were checked for spelling errors and regionally differing synonyms. Groups of species were renamed to their genus if possible, or alternatively renamed to the following groups: conifers, short-lived broadleaves or long-lived broadleaves. Short-lived broadleaves included early-colonizing and fast growing species like *Populus*, *Alnus* and *Salix* as well as all species that usually do not grow into the main canopy layer (*Sorbus*, *Prunus*), while long-lived broadleaves included slower growing (often late successional) species like *Quercus*, *Fraxinus*, *Castanea*, *Tilia*. As a result, we obtained a list with 165 names of species and groups of species, with very different frequency of occurrence. To obtain sufficiently large groups for our analysis, we iteratively grouped these at increasing taxonomic level, using the hierarchy subspecies–species–genus–other conifers/other short-lived broadleaves/other long-lived broadleaves. Additionally, we merged the species *Quercus robur* L. and *Quercus petraea* (Matt.) Liebl. into one group since not all NFIs separated these species. Species or groups were retained if they had at least 1000 tree observations. This process resulted in 41 species and species groups. Next, we determined the shares of each species in the number of observations in each region as well as for all regions combined. We kept those that had at least a 5% share overall, or at least 10% of the number of tree observations in at least one region. As a result, we identified 10 individual species, the group *Q*. *robur + Q*. *petraea*, the genus *Betula* spp. and the three groups conifers/short-lived broadleaves/long-lived broadleaves for our analysis (Table 2).

**Table 2:** Share of each species (group) in the total basal area (DBH ≥ 120 mm) per region at first measurement (%)^1^.

	Boreal			Continental/Alpine				Atlantic				Mediterranean	
	Northern Finland	Southern Finland	Småland	Bavaria	Alps	Jura+ Plateau	Maribor	Netherlands	North Rhine-Westphalia	Southern and Eastern Ireland	Galicia	Catalonia	Castilla y Léon
*Abies alba* Mill.				2	10	17	8					2	
*Picea abies* L. (H. Karst)	24	34	45	52	52	35	28	4	44	4		0	0
*Picea sitchensis* (Bong.) Carr.								0	0	59			
*Pinus halepensis* Miller												15	0
*Pinus nigra* J.F. Arnold				0	0	0	0	6	0	1	0	13	3
*Pinus pinaster* Aiton								1			34	2	36
*Pinus sylvestris* L.	61	47	38	20	3	4	8	32	7	1	0	24	27
Other conifers	0	0	0	3	11	2	1	14	4	14	2	14	8
*Betula* spp.	14	13	10	1	1	0	1	5	3	3	1	0	0
*Eucalyptus globulus* Labill.											12	0	
Other short-lived broadleaves	1	4	4	2	2	1	3	5	4	6	2	2	2
*Fagus sylvatica* L.			0	11	11	27	27	6	18	3	0	4	5
*Quercus ilex* L.							0				0	9	6
*Quercus robur* L. + Q. petraea (Matt.) Liebl.		0	3	5	1	4	9	18	13	5	19	2	2
Other long-lived broadleaves	0	1	0	4	9	10	15	9	6	4	30	13	11
Total	100	100	100	100	100	100	100	100	100	100	100	100	100

1: An empty cell means the species is not present, 0% means a share of less than 0.5%.

All trees were assigned to a 5-cm DBH class using the DBH at the first measurement. Original ownership classes were recoded into public/private. Forests in cooperative ownership were considered to be private. All trees were labelled as being harvested or not, and as dead or not. Whenever present, the class “dead, harvested” was labelled as “not dead” and “harvested” for reasons of consistency among the regions. This class was present for 21 sample trees in Southern Finland, 3 sample trees in Northern Finland, 446 sample trees in the Alps region and 656 trees in Jura+Plateau. The class “dead” thus represents trees that died between the inventories and were still present in the inventory plot at the second measurement. In the Spanish regions no distinction was made between harvested trees and lying dead trees, these were all labelled as “harvested”. Standing dead trees were available as a separate class and labelled as “dead”.

### Data analysis

We computed annual probabilities for a tree being harvested and for a tree being dead using the following formula:

$$z=1-{\left(1-\frac{\sum M_{h}}{\sum M}\right)}^{\left(\frac{1}{X}\right)}$$ (2)

where *z* is the annual probability that a tree of a certain population is respectively harvested or dead, *M* the number of live trees of that population in the first measurement, and *M*_*h*_ the number of trees of that population that respectively have been harvested or that died between the first and the second measurement. A population can consist of any combination of species, owner class and/or DBH class. The measurement interval X was calculated as the mean value over all plots in the region (Table 1). In case of calculating probabilities over multiple regions, a weighted mean was calculated using the total number of trees per region as weight.

Our analysis consisted of two parts. The first was a general comparison across regions, species and owners, and the second was an inspection of the harvest and mortality patterns over DBH for these groups. For the general comparison we only included trees equal to or larger than 120 mm DBH, as this was the highest threshold of all NFIs included. Per region we computed the share of total basal area per species at the time of first measurement, the quadratic mean DBH for all live trees at the first measurement, the harvested trees and the trees that were found dead, for the region as a whole and per species. Furthermore, we computed the annual harvest and mortality probability as described above for all combinations of species and owners within a region, and for all species across regions. We used a Chi square test of independence to test for differences between owners within a region, calculating χ^2^ as:

$${\chi^{2}}_{j}=\sum_{o}^{O}\sum_{h}^{H}\frac{{\left(n_{ohj}-\frac{n_{oj}n_{hj}}{n_{j}}\right)}^{2}}{\frac{n_{oj}n_{hj}}{n_{j}}}$$ (3)

with χ^2^_j_ the χ^2^-statistic for region *j*, *O* the total number of owners, *H* the status of trees as either harvested or not harvested, *n*_*oh*j_ is the number of trees observed with ownerclass *o* and harvest status *h* in region *j*, *n*_*oj*_ the total number of trees observed for ownerclass *o* in region *j*, *n*_*hj*_ the number of trees observed with harvest status *h* in region *j* and *n*_*j*_ the total number of trees observed in region *j*. With *O* and *H* being 2 in all regions, this yields a test statistic with 1 degree of freedom.

For the second part of the analysis, the inspection of patterns over DBH, all available observations were included. Annual harvest and mortality probabilities were computed per DBH class for all species in all regions, for all owners together as well as per owner class. Probabilities were only computed if at least 10 observations were available. For a subset of species and region combinations we compared the regional DBH pattern found with the handbook intensities as defined by growth and yield tables. For each of the groups in the subset we selected a growth and yield table publication best matching the conditions in the region. For all entries in the tables, for all site classes available, we computed the harvest intensity according to Eq 2, with M the number of trees per ha (stand density) before thinning and M_h_ the number of trees per ha thinned, and X the age interval since the previous entry in the table. We attributed the harvest intensity to a DBH class using the diameter before thinning. From the resulting set of DBH class-harvest intensity pairs we selected for each DBH class the lowest and highest harvest intensity to represent the range of harvest intensities as recommended by the handbook.

## Results

### Results by regions

Most regions have around 10 species present, of which 4–6 have a share higher than 5%, except the northern regions with only 3 major species (Table 2). The dominant species in all regions is a coniferous species. For 6 regions this is *Picea abies* L. (H. Karst), for one region *Picea sitchensis* (Bong.) Karr., for 4 regions *Pinus sylvestris* L. and for 2 regions *Pinus pinaster* (Aiton). Only Galicia and Maribor have a total coniferous share of less than 50%. *Quercus* spp. and *Fagus sylvatica* L. are often the most important broadleaves, except in the Northern regions where *Betula* spp. fulfils this role. The group “other long-lived broadleaves” also makes up ca. 10% or more of trees in about half of the regions, while “other short-lived broadleaves” are less frequent.

**Fig 2:**
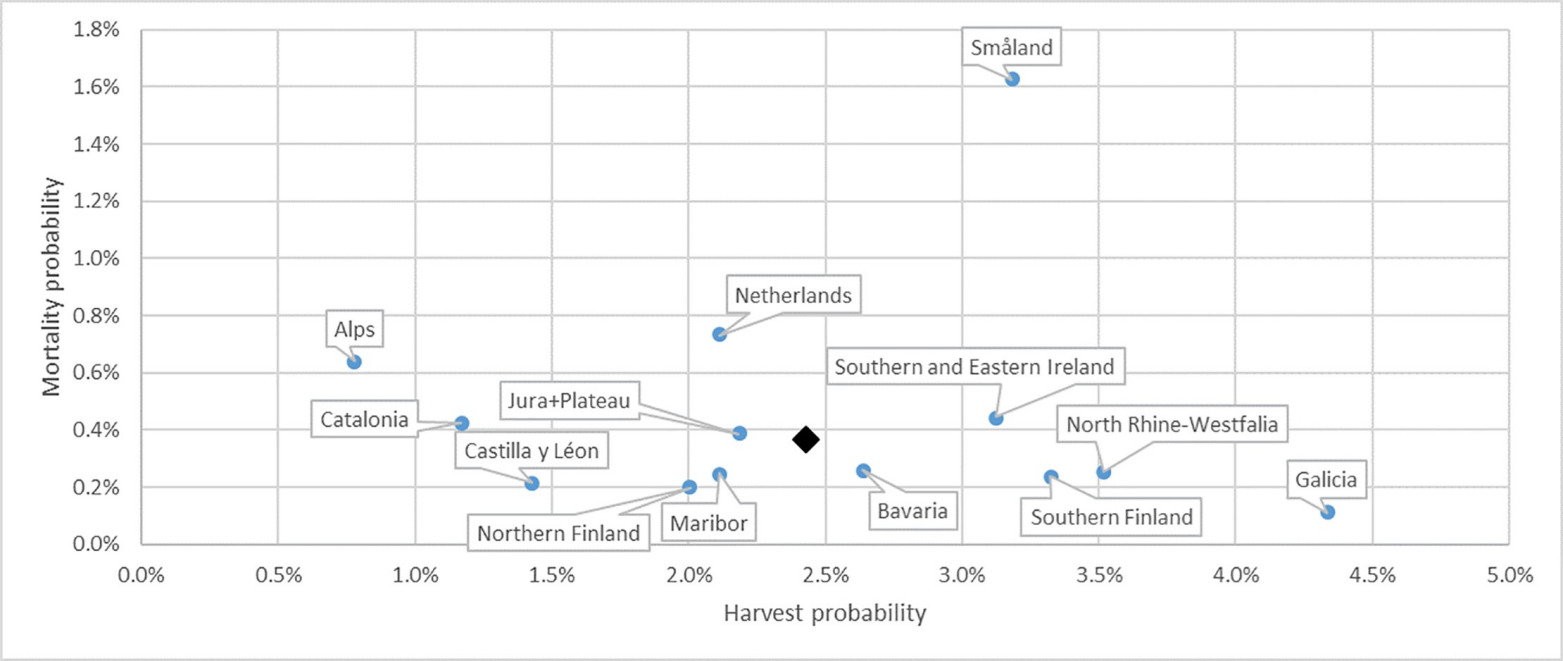
Annual mortality probability versus harvest probability, by region over all investigated species and diameter classes, using a common DBH threshold of 120 mm. The black diamond indicates the average over all regions.

**Fig 3:**
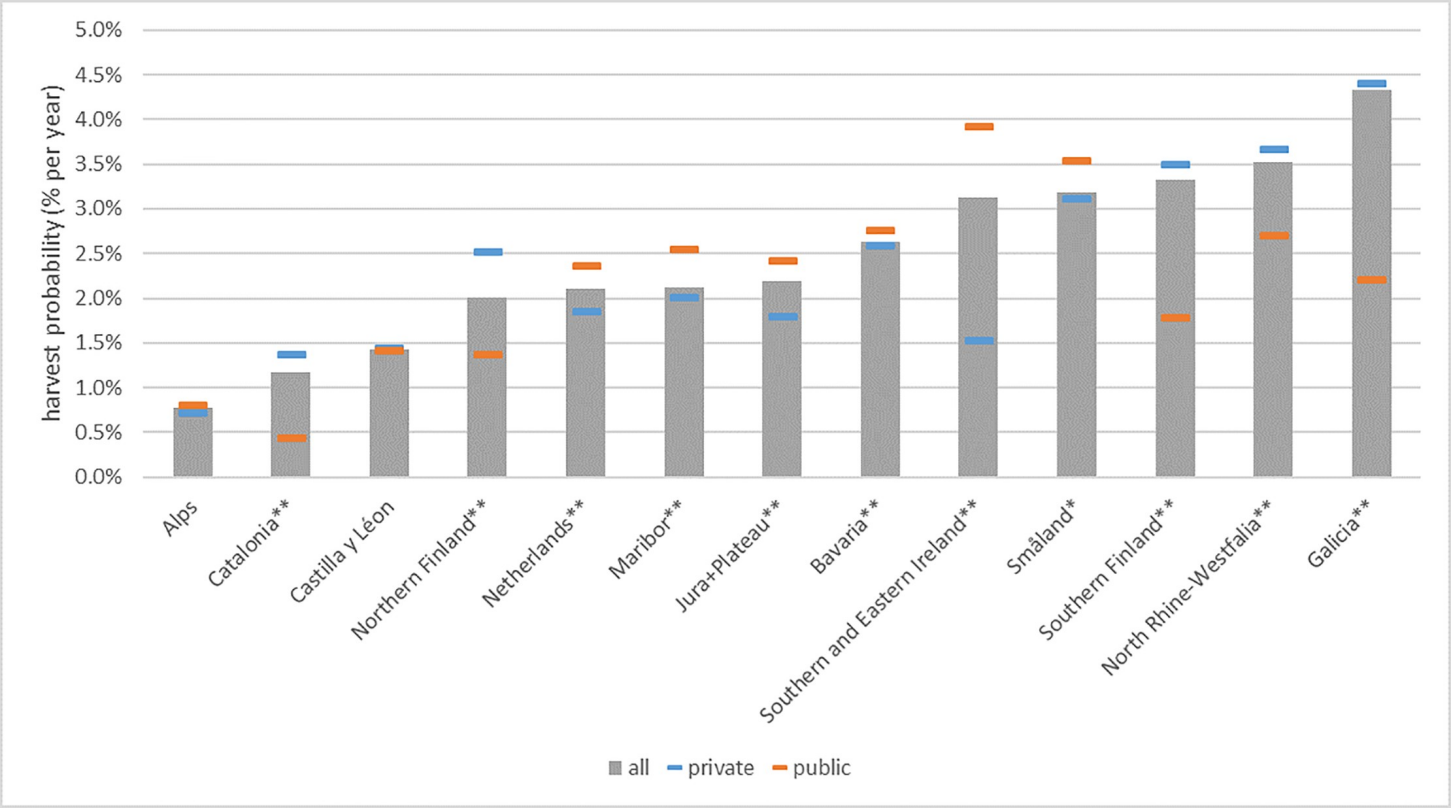
Annual harvest probability, by region and owner class, using a common DBH threshold of 120 mm. Stars indicate a significant difference between private and public owners (one star p<0.01, two stars p<0.001).

The average probability of a tree being harvested over all regions is 2.4% per year, with the highest probability in Galicia (4.3% per year) and the lowest in the Alps (0.8%) (Fig 2). For most regions the probability of a tree being dead on site is below 0.45%, except for the Alps, the Netherlands and Småland, with Småland far above the other regions (1.63%). The average mortality probability is 0.4% per year. There is no clear tendency for increased mortality with decreasing harvest pressure. For most regions, there is a significant difference in harvest probability between public and private owners (Fig 3): six regions out of 13 have a higher harvesting probability in publicly owned forests. However, in the Finnish regions, in North Rhine-Westphalia and in Catalonia the opposite was observed.

**Fig 4:**
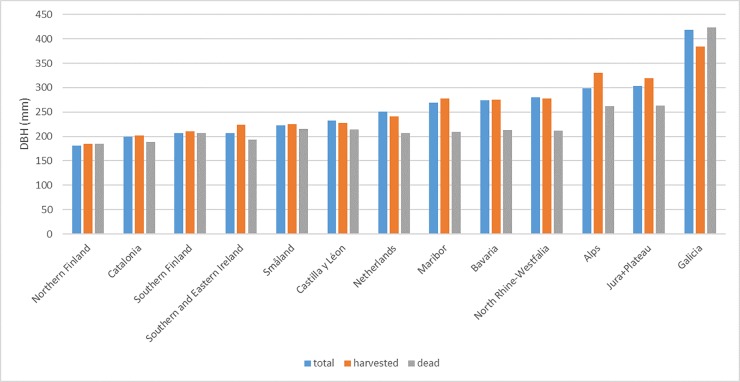
Quadratic mean DBH of the total population at first measurement, of harvested trees and of dead trees by region, using a common DBH threshold of 120 mm.

The quadratic mean DBH is between 180 and 250 mm in the boreal and Mediterranean biogeographic region and between 250 and 300 mm in the continental/alpine biogeographic region (Fig 4). The Atlantic biogeographic region shows a great spread with only 210 mm for Southern and Eastern Ireland and 420 mm for Galicia. The quadratic mean DBH of the dead trees is in almost all regions below the quadratic mean of the total population, except for the Finnish regions and Galicia. There is less of a pattern if we compare the quadratic mean DBH of the harvested trees with the total population. Some regions seem to target the larger trees for harvesting (Alps, Jura+Plateau, Southern and Eastern Ireland), while Galicia clearly shows the opposite pattern. However, in most regions the differences are only minor.

### Results by species

**Fig 5:**
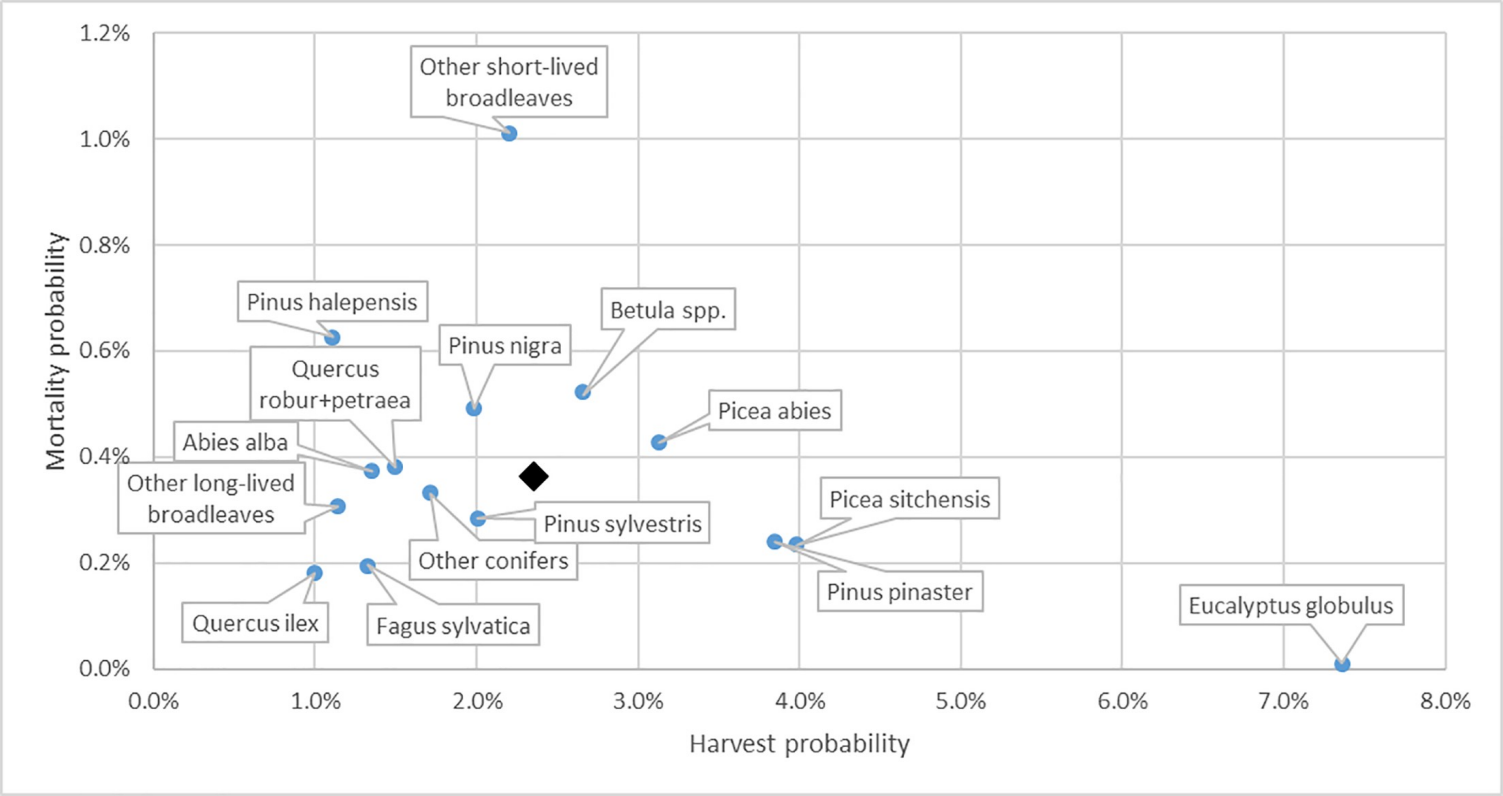
Annual mortality probability versus being harvest probability by species across all regions, using a common DBH threshold of 120 mm. The black diamond indicates the average over all species.

**Fig 6:**
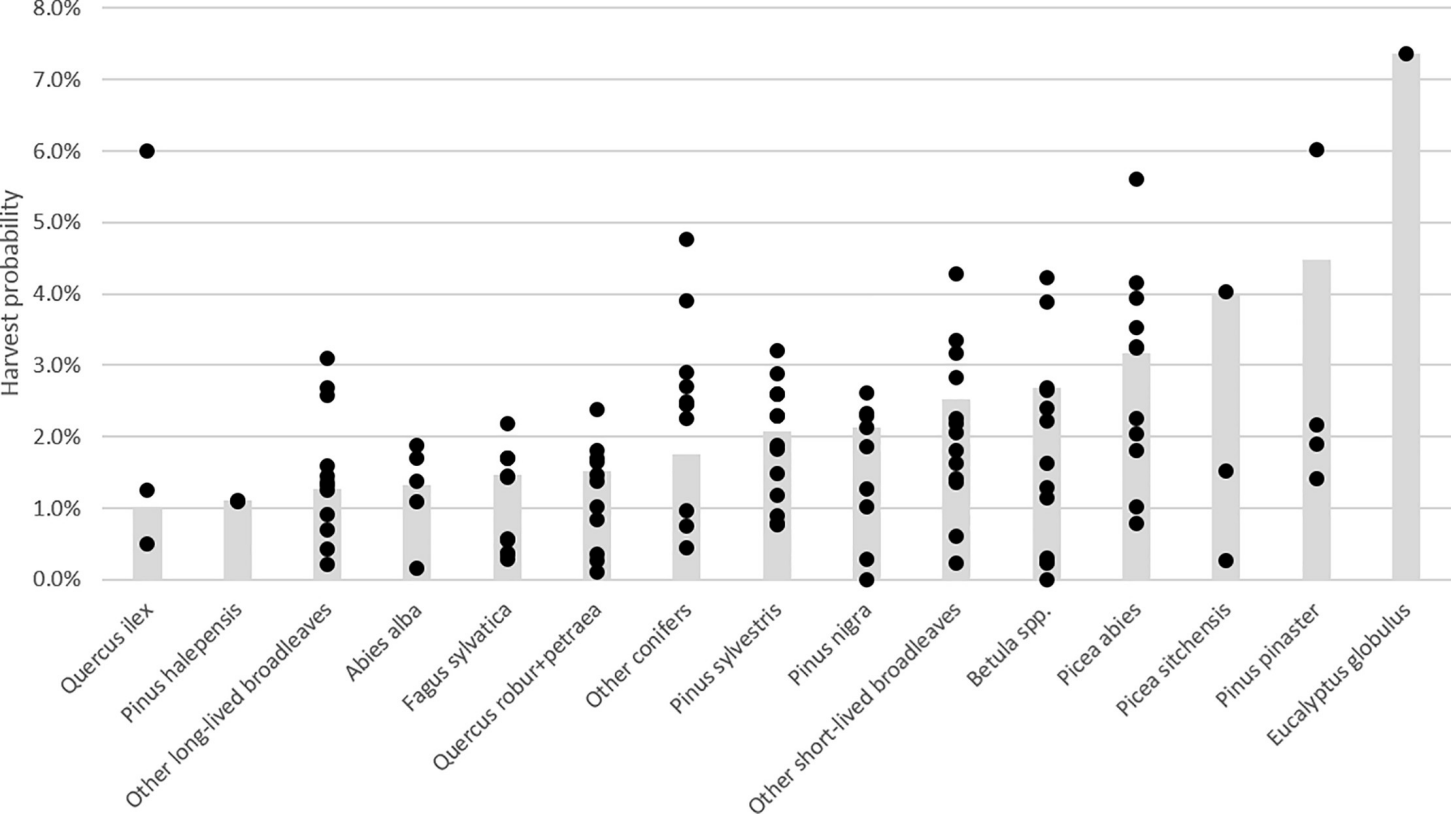
Annual harvest probability by species, over all regions (bars), and for individual regions (black dots). Values by region and species can be found in S1 File.

The harvest probability per species across all regions ranges from 1.0% (*Quercus ilex* L.) to 7.4% (*Eucalyptus globulus* Labill.) (Fig 5). The spread in harvest probability per species over the regions is large (Fig 6, S1 File). Most species are hardly harvested in at least one region, and are intensively harvested in at least one other region. The average mortality probability ranges from 0% (E. globulus) to 1.0% (other short-lived broadleaves), with most species in the range 0.2%-0.6% (Fig 5, S1 File).

### Results by DBH classes

**Fig 7:**
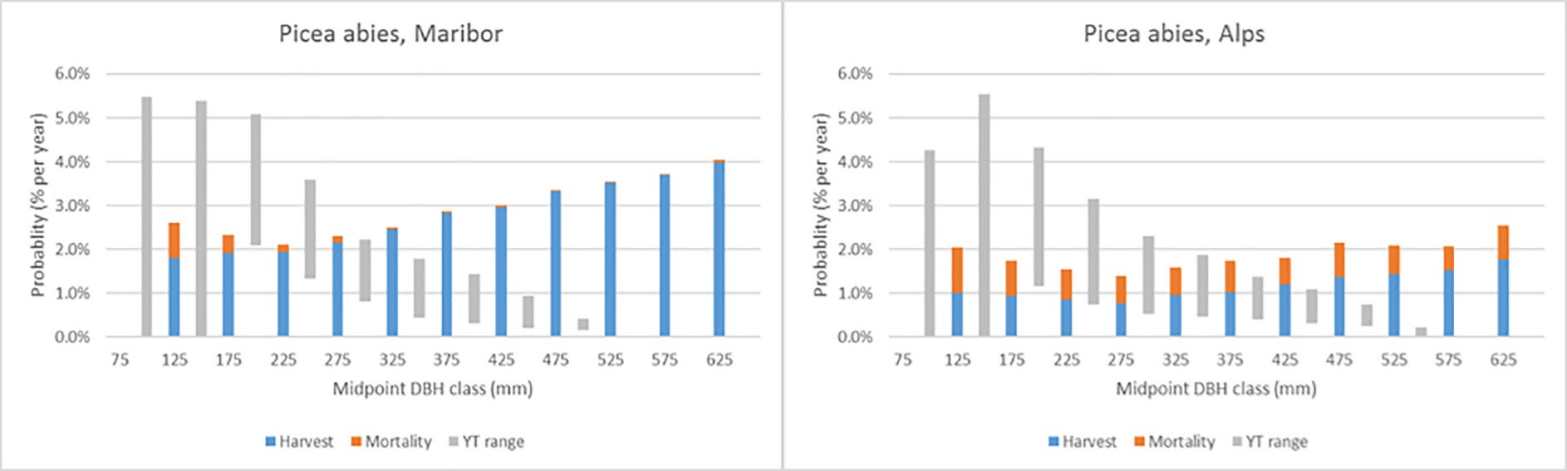
Annual actual harvest and mortality probabilities (% per year) per DBH class (mm) for *P. abies* in two regions and the yield table (YT) range of being harvested.

**Fig 8:**
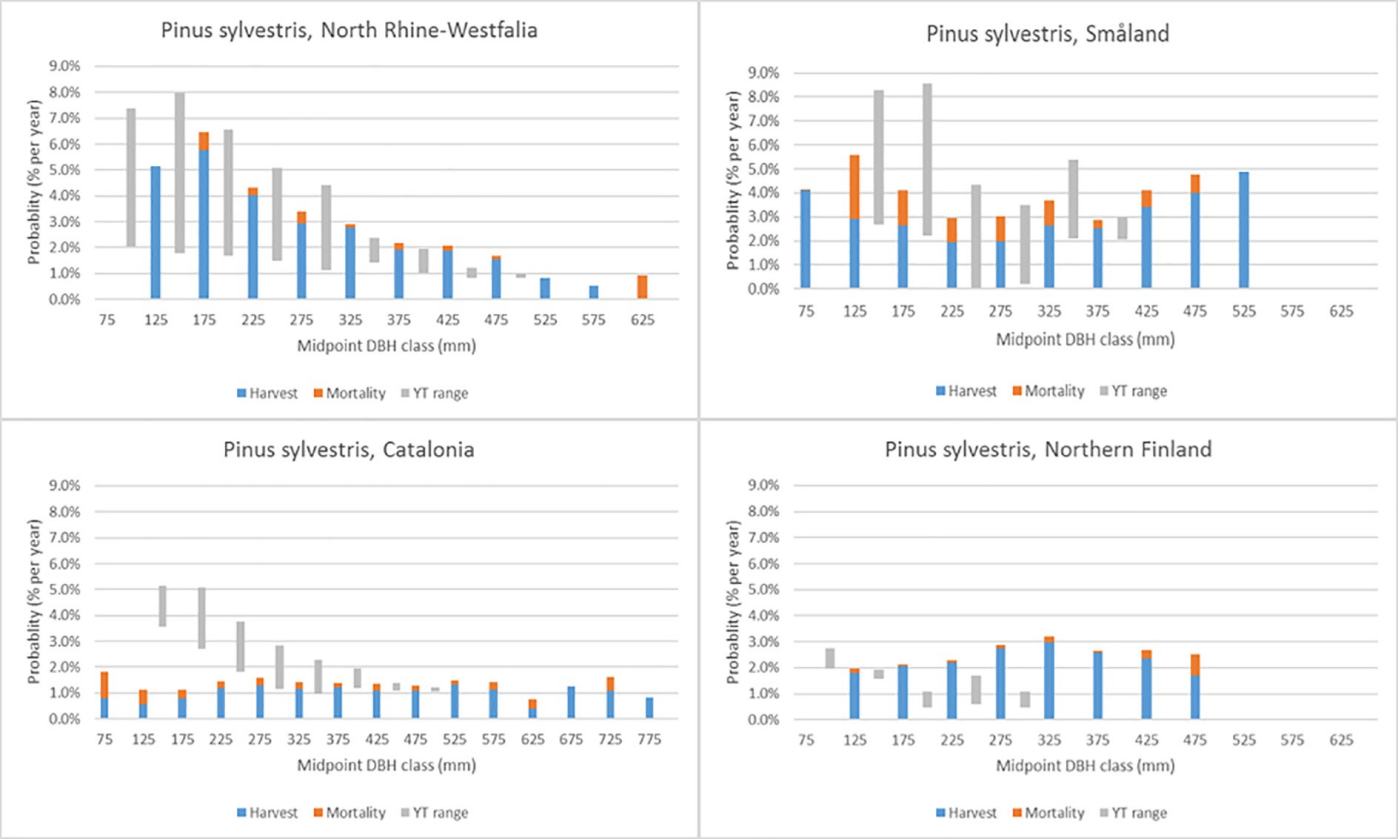
Annual actual harvest and mortality (% per year) per DBH class (mm) for *P*. *sylvestris* in four regions and the yield table (YT) range of being harvested.

**Fig 9:**
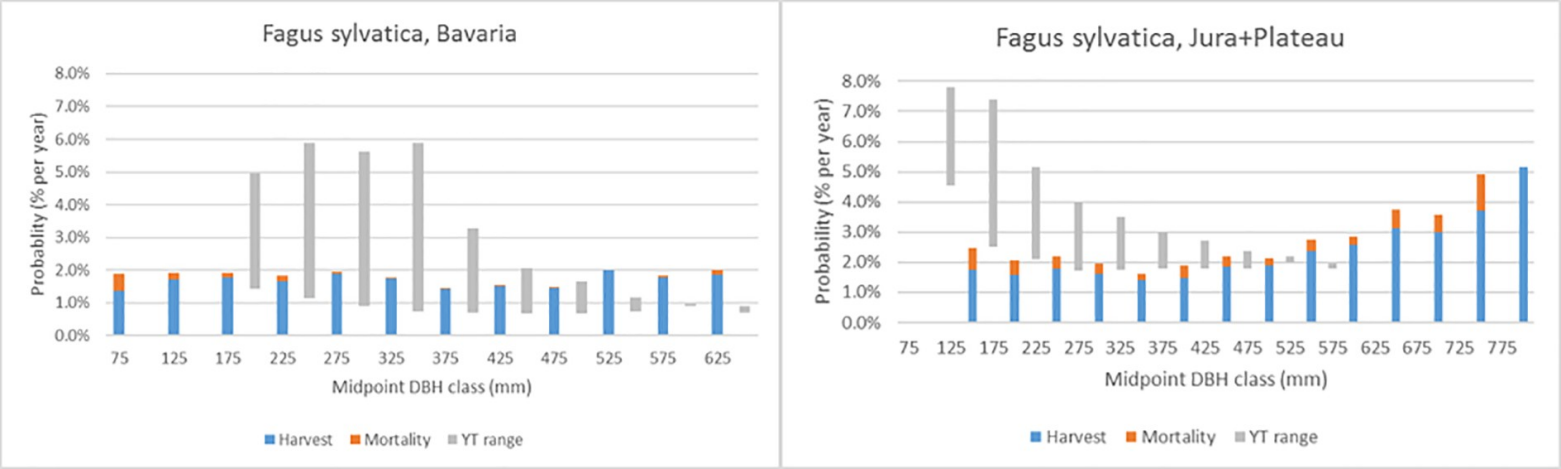
Annual actual harvest and mortality probabilitis (% per year) per DBH class (mm) for *F*. *sylvatica* in two regions and the yield table (YT) range of being harvested.

**Fig 10:**
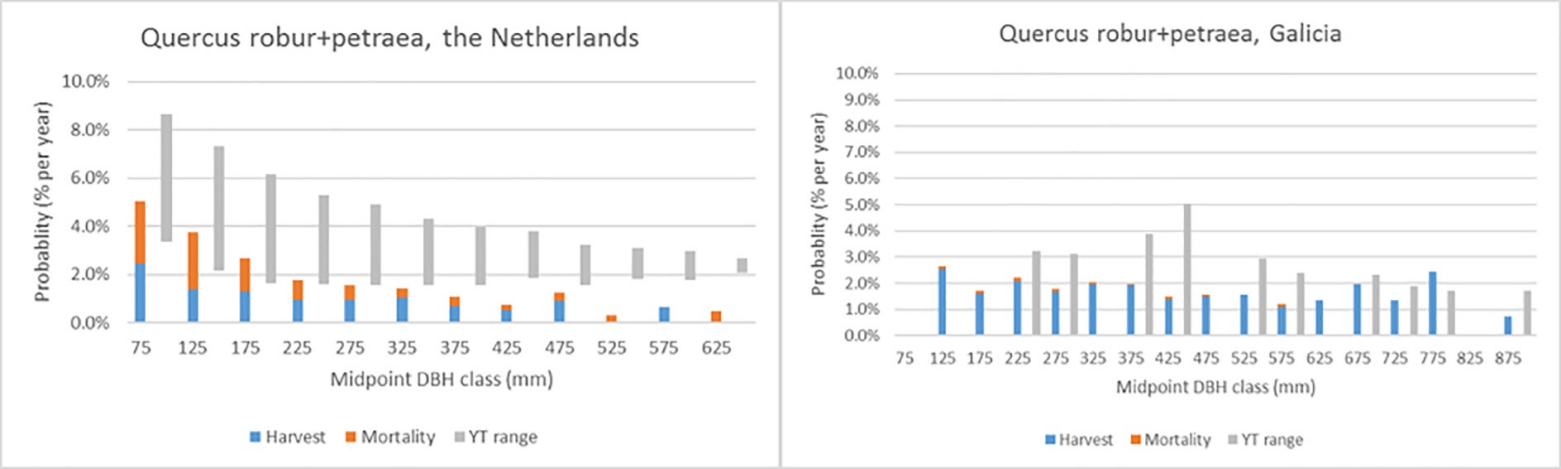
Annual actual harvest and mortality probabilities (% per year) per DBH class (mm) for *Q*. *robur + Q*. *petraea* in two regions and the yield table (YT) range of being harvested.

**Fig 11:**
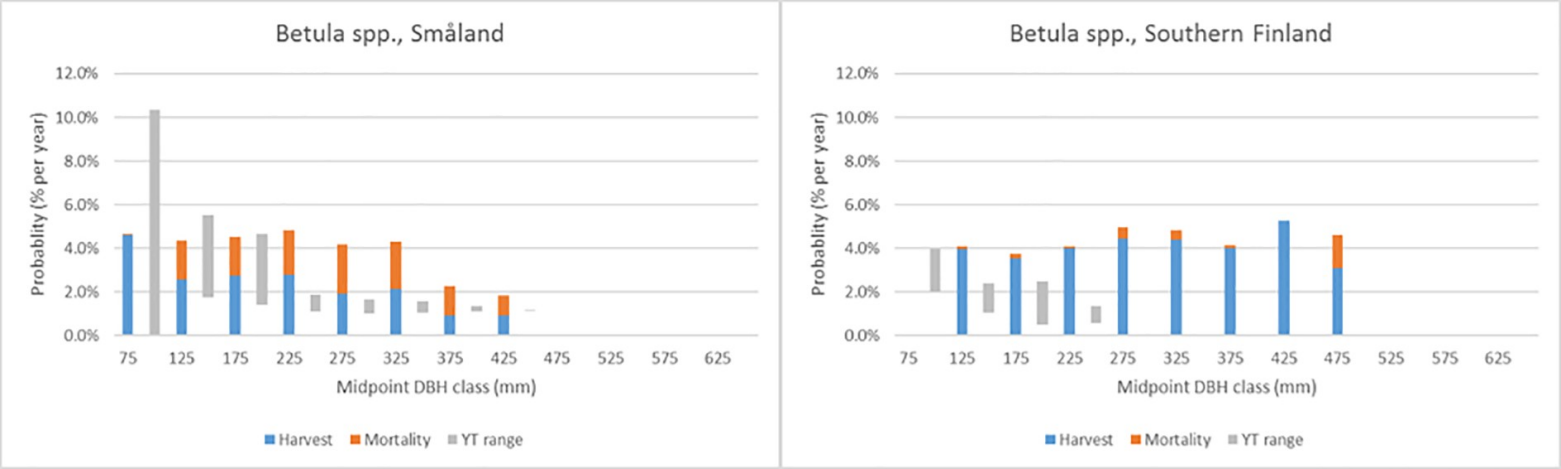
Annual actual harvest and mortality probabilities (% per year) per DBH class (mm) for *Betula* spp. in two regions and the yield table range (YT range) of being harvested.

Figs 7–11 demonstrate the variety of harvest and mortality patterns over DBH for the two most common coniferous species and the three most common broadleaves, and compare these patterns with the patterns as calculated from growth and yield tables representing ‘handbook harvesting intensity’. See Table 3 for the growth and yield tables that were selected for the comparisons. S1 Fig. shows the same graphs for the four most common species per region, while S2 File contains all graphs for all species in all regions, separately by private and public owners, as well as for all owners together. Shade-tolerant conifers such as *P*. *abies*, *P*. *sitchensis* and *Abies alba* Mill. mostly show an increasing harvest probability with DBH (similar to *P*. *abies* in Maribor, Fig 7), but in some regions the shape is more flat (*P*. *abies* in Alps, Fig 7). The more light-demanding *Pinus* species mostly show a flat or decreasing harvest probability with DBH, but other shapes are found as well (Fig 8). *F*. *sylvatica*, the *Quercus* species and the group “other long-lived broadleaves” often show a relatively flat harvest pattern over DBH (Figs 9 and 10), but for most continental/alpine regions (Alps, Jura+Plateau, Maribor) for *F*. *sylvatica* the harvest probability increases with DBH. *Betula* spp. and the group “other short-lived broadleaves” tend to show flat to decreasing harvest probabilities with DBH. In most cases, the patterns we found are clearly different from those derived from the growth and yield tables.

**Table 3:** Growth and yield tables used as comparison for handbook harvest intensity.

Species	Region	Region of origin of growth and yield table	Reference
Picea abies	Maribor	Austria (Bruck-Mur)	Marschall 1975 [40]
	Alps	Austria (Hochgebirge)	Marschall 1975 [40]
Pinus sylvestris	North Rhine-Westphalia	Germany	Wiedemann 1949 [41]
	Småland	Sweden	Andersonn 1962 [42]
	Catalonia	Spain (Pyrenees)	Garcia Abejon 1986 [43]
	Northern Finland	Northern Finland	Koivisto 1959 [44]
Fagus sylvatica	Bavaria	Germany (Lower Saxony)	Schober 1967 [8]
	Jura+Plateau	Switzerland	Badoux 1983 [45]
Quercus robur+petraea	Netherlands	Netherlands	Jansen 1996 [46]
	Galicia	France	Bisch 1987 [47]
Betula	Småland	Norway	Braastad 1996 [48]
	Southern Finland	Southern Finland	Koivisto 1959 [44]

## Discussion

### Regional patterns

#### Boreal regions

There is an increasing boreal gradient from Småland via Southern Finland to Northern Finland, visible as an increasing share of *P*. *sylvestris* and *Betula* spp., and a decreasing share of *P*. *abies*. Småland and Southern Finland are both on the high end of harvest probabilities, while in Northern Finland it is below average. This reflects the generally intensive forest management in Nordic countries, and the less favourable growing conditions in the most northern part. Most forests in Småland and Southern Finland are privately owned, while in Northern Finland public and private have about equal shares. In line with the general pattern, public forests in Småland are managed more intensively than private forests. However, in both Finnish regions the opposite is found. An important reason is forest protection: 33.8% of publicly owned forest land in Finland is outside wood production, against only 0.6% in private forests [49]. The boreal regions have relatively low mortality in low DBH classes (especially the Finnish regions), probably due to low tree densities when planting, more attention to early thinnings and thus less competition-induced mortality. The harvest probability over DBH shows a relatively flat pattern, reflecting the intensive tending of stands.

Results for Småland in this study are highly affected by the storm Gudrun that hit the region in January 2005 and felled approximately 70 million cubic meters of timber [50]. This was almost as much as the average annual cut for the whole of Sweden and three times the annual cut in southern Sweden. Mainly recently thinned stands dominated by P. abies were affected. Mortality probability in Småland is by far the highest of all regions included in the study and also harvesting probability is among the highest. This can be explained both by the general high logging intensity in the region with a very active forestry, but also that undamaged trees had to be harvested in order to facilitate sanitation logging, and to stabilise the new forest edges.

#### Continental/Alpine regions

All continental/alpine regions have a high share of *P*. *abies*, a reasonable share of *F*. *sylvatica* and feature the presence of *A*. *alba*. Only Bavaria has a sizable share of *P*. *sylvestris*, present in the lower altitudes. The Alps region stands out from the other regions due to its low harvest probability, rather high mortality (in all DBH classes) and no significant difference between public and private owners. Harvests in mountain regions are only rarely profitable due to steep terrain, poor accessibility and high labour costs, particularly in Switzerland. Management in these conditions is to a large degree driven by regulations and subsidies for management of forests that are primarily managed to protect against avalanches, rockfall and landslides [13]. Forests in the other regions are more accessible, with harvest probabilities close to average. Overall, harvesting is more concentrated in the higher DBH classes and public forests. Mortality rates are clearly influenced by sanitation fellings (removal of trees that are unhealthy, for example infested by bark beetles) and salvage loggings (removal of dead trees after fire or storm events). If we include the salvaged trees (recorded as dead and harvested) in the mortality estimates for the Swiss regions, mortality in Jura+Plateau increases from 0.39% to 0.63%, and in the Alps from 0.64% to 0.75%. In Maribor the overall mortality is very low and almost absent in the middle and higher DBH classes, also a sign of intensive salvage logging. Only in Bavaria there is some tendency of increasing mortality in higher DBH classes for the most common species, while these are found only for a few species in the Swiss regions.

#### Atlantic regions

The Atlantic regions vary greatly in tree species composition and harvest intensity. North Rhine-Westphalia (NRW) has better growing conditions with a high share of *P*. *abies* (44%) and *F*. *sylvatica* (18%), while in the Netherlands the most important species are *P*. *sylvestris* (32%) and *Q*. *robur + Q*. *petraea* (18%), growing on poor sandy soils. This is reflected in the harvest probability, with North Rhine-Westphalia having the second highest harvest probability of all regions in this study (3.5%) and the Netherlands below average. An important contributor is *P*. *abies*, with 5.9% the highest harvest probability of P. abies in all regions. During the observation period NRW was severely hit by storm Kyrill (2007), causing damage equal to about three times the normal annual logging [51]. The observed high harvest probability, especially in private forests, is to a large extent a result of the storm damage and subsequent clear-up. In general the harvest intensity in Germany is lower for private forests than for state forests: 60% of NRW’s forests are privately owned, and small scale structures are dominant [52]. In the Netherlands, nature conservation is important for both private and public owners. About 40% of the forest is designated to nature conservation, while the other 60% is usually managed as multi-purpose forests [53], with the shares rather equal for public and private forests. The lower harvest probability in private forests is mostly attributed to inactive owners. The Netherlands has an active subsidy scheme with requirements for the amount and size of dead wood present in the forest (both in nature and multi-functional forests), which is probably the reason for the higher mortality compared to other regions, and the occurrence of mortality in all DBH classes. On the contrary, in North Rhine-Westphalia mortality is low and concentrated in the smaller trees, an indication of active removal of dead trees.

The regions Southern and Eastern Ireland and Galicia share a high probability of harvesting. Both regions show a very sharp distinction in plantation species and non-plantation species. Southern and Eastern Ireland has a share of 59% of *P*. *sitchensis*, with a harvest probability of 4.0%, while especially the broadleaves are hardly managed (all below 1.0%). The main plantation species in Galicia are *E*. *globulus* and *P*. *pinaster*, with a share of respectively 12 and 34%, and harvest probabilities of 7.4% and 6.0%. About half of the forests consists of *Q*. *robur + Q*. *petraea* and long-lived broadleaves (mostly *Quercus pyrenaica* Willd. and *Castanea sativa* Mill.) with much lower harvest probabilities (1.5–1.6%). Despite this, the mortality in both regions is very low, also in the less-harvested broadleaved species. Mortality is rather equally distributed over DBH classes in both regions, with the consequence that the average DBH of dead trees in Galicia is about equal to the average of the total population, while for most regions dead trees are clearly smaller. There is a huge difference in harvest probability between public and private forest owners in Southern and Eastern Ireland. Most of the private owners are farmers that have availed of Exchequer and EU funded support schemes to afforest their lands in the last 2 decades [54]. Many of these forests are still young but many are now at a stage where they could be thinned, and it is clear that it is difficult to engage private owners in the active management of their forests [55].

#### Mediterranean regions

The tree species distribution of Catalonia and Castilla y Léon reflect their southern locations. Besides a ~25% share of *P*. *sylvestris*, there are typical Mediterranean pines like *P*. *halepensis* and *P*. *pinaster*, and a sizable share of *Q*. *ilex* and other long-lived broadleaves. The latter group is dominated by Mediterranean oaks like *Quercus suber* L., *Q*. *pyrenaica*, *Q*. *faginea* Lam. and *C*. *sativa*. Both regions have a low harvest probability (1.2% and 1.4% respectively for Catalonia and Castilla y Léon), only the Alps region is lower. A large share of the public forests in Catalonia consists of National and Natural Parks, located in the Pyrenees and other mountainous areas. They have a very low harvest intensity with management focussed on the provisioning of ecosystem services such as soil protection against erosion, water regulation and recreation. As a consequence, there is a large difference in harvest intensity between public and private forests in Catalonia. In contrast, we found no difference in harvest intensity in Castilla y Léon between public and private owners. Mediterranean forests are often irregular in structure and age, and in many cases managed through selective fellings with long intervals [56,57]. This is reflected in the flat distribution of harvest probability over DBH, and mortality in all DBH classes. Only *P*. *pinaster*, the most important species in Castilla y Léon, shows signs of a more plantation-type of harvesting: an increased harvest probability at higher DBH, and a higher overall harvest probability. Mortality is overall higher in Catalonia (0.4%) than in Castilla y Léon (0.2%), and shows in Catalonia signs of a U-shape for some species. Both harvesting and mortality are heavily influenced by fires: if we exclude the plots within the perimeters of the severe fires of 1994 and 1998 in Catalonia, the overall harvest probability decreases from 1.2% to 0.3%, and the mortality probability from 0.4% to 0.2%. Also drought and heatwave events are known to be important contributors to mortality in the region [58], but their effects cannot directly be assessed with our dataset.

### Harvest patterns

Harvest probability greatly varies among owners, regions and species. There is a general trend for higher harvest probability in regions with higher productivity and faster growing species, but this is mediated by a number of factors. In the Alps region, difficult accessibility and a focus on the protection function lowers the harvest intensity, while the boreal regions may have a higher intensity than expected, due to a strong forestry tradition and facilitated by a vast resource. Ownership is another important factor. In many regions, privately owned forests have a lower harvest probability than publicly owned forests. Generally, this is attributed to the fact that there are many private owners with only small properties, often with little knowledge of forest management, and not living on or close to their forest property [59–62]. A positive relationship between harvesting intensity and property size is among others reported by Beach et al. [20]. Strong forest owners associations or other ways of organising common ownership or management, as for instance in Småland and Bavaria, is a way to promote harvesting also for small private forest owners [63]. In Southern and Eastern Ireland, lower harvesting probabilities in privately owned forests are partly caused by the fact that most of these forests have been planted only recently. In the regions where public forest is managed less intensively than private forest, this is usually caused by a strong focus of the publicly owned forests on nature conservation, often through the ownership of National and Natural Parks like in Catalonia and the Finnish regions. In other regions, National Parks are privately owned (Netherlands), or public forest services do not only manage natural parks but also manage large areas of production forests. The regional patterns we found are well in line with those found in [64]. They found a strong correlation of harvest intensity with forest-resource related variables such as the share of plantation species, site conditions (i.e., topography, accessibility), and country-specific characteristics, which is confirmed by our study. They found less influence of socio-economic variables, which may be explained by the different focus of public forest owners in different regions.

Different types of harvesting are reflected in the patterns we found. Regions and species with predominantly selective cuttings have a low harvest probability and flat (Catalonia) or increasing (Alps) harvest probability over DBH. Plantation species usually have a high harvest probability, increasing with DBH, and are often shade-tolerant conifers. Light-demanding conifers such as pines tend to have a medium harvest probability, decreasing with DBH. Only in regions where they are managed more intensively do they show a U-shape or flat/increasing tendencies (*P*. *sylvestris* in the boreal regions, *P*. *pinaster* in Castilla y Léon and Galicia). *Quercus* species, F. *sylvatica* and other long-lived broadleaves usually have low harvest probabilities, with *F*. *sylvatica* having a flat or increasing distribution over DBH, and the other species a flat or decreasing distribution. *Betula* spp. and other short-lived broadleaves are not very common in most regions and patterns are flat or irregular. It is unclear to what degree patterns over DBH are determined by species identity and traits (light demanding versus shade tolerant), and to what degree by intensity of (plantation-like) harvesting. The patterns we found do not agree with the handbook intensities from the growth and yield tables in most cases. Often the actual harvest probability in low DBH classes is lower than prescribed, and higher in higher DBH classes. This may be related to planting less trees than prescribed in the regeneration phase, but also to a tendency to avoid thinnings that are not commercially viable.

### Mortality patterns

Harvesting and natural mortality are closely interlinked. Generally, forest harvesting is expected to reduce natural mortality. This can be directly, through targeting smaller trees that are likely to die from competition (thinning from below) or those that are vulnerable to natural hazards (final harvest of trees before they are so tall that they suffer from wind damage, reduction of fuel load to reduce wildfire risk), or indirectly, through increasing the growing space of the remaining trees and by harvesting the trees before they reach the end of their life span. Conversely, the occurrence of natural mortality can also trigger harvesting activities, usually in the form of salvage logging. Salvage logging is usually applied after the occurrence of natural disturbances, and is practiced to recover some monetary value of the trees that are lost, and/or to prevent the subsequent outbreak of insect attacks, but also to facilitate scarification and planting of heavily damaged stands. As a consequence,”pure” harvest probabilities in our analysis will be inflated, while “pure” mortality probabilities are underestimated. The Swiss and Finnish NFIs are the only inventories that explicitly indicate if a tree was dead before it was harvested, but in many cases this is nearly impossible to judge, especially with long census intervals. This is testified by the fact that in the code “dead, harvested” was hardly used in the Finnish cases. However, the Swiss data gives us a first estimation of the order of magnitude, together with the analysis of known extreme events like the fires in Catalonia and the storm damage in Småland. Most regions show strong indications of salvage logging, visible as low mortality probability regardless of harvest intensity, and the almost complete absence of mortality in mid to high DBH classes. This observation is also supported by the fact that especially *P*. *abies* in Central Europe greatly suffers from bark beetle attacks [65–67], but this is not visible in the mortality patterns. Indeed, most countries have strict regulations about the removal of damaged trees [68,69].

Studying natural mortality rates and patterns is a challenge in this dataset, due to the fact that dead trees are usually removed. Mortality rates are therefore generally lower than reported in other studies. Neumann et al. [70] found a mean annual mortality rate (the average percentage of trees dying per year across all plots) of 0.50% per year in Europe’s forests, on a set of trees that was assessed annually. Further work is needed to explore the interlinkages between mortality and harvesting in this dataset, and the patterns found. Further studies should perhaps consider wider DBH classes and/or make more targeted observations, as for example was done in Lorimer et al. [71].

### Harmonisation

Although NFIs rely on the same principles, important differences exist in terms of definitions, sampling design and intensity, plot design, thresholds and for our database also for recording year (e.g. Galicia data cover 1986–1998, while Swedish data covered 2005–2014) [72]. When comparing between regions, we harmonised our dataset with regards to DBH threshold and species, owner groups, status of dead or harvested, and we corrected for sampling probability in relation to size and for different census intervals. We tried to balance the number of trees sampled per region to account for different sampling intensities among regions. Harmonisation is needed to ensure comparability among regions but leads to a loss of data and information in regions where measurements are more detailed. For example, the application of the 120 mm DBH threshold leads to a reduction of 10.8% in number of observations that we could use. However, the same type of analysis can be applied to individual regions, with the possibility to include a larger diversity of species and owner classes, and with the local DBH threshold. In the light of our analysis, we would advise NFIs to make a clear distinction between trees that are harvested and those that remain dead on site. In Spain for example, no distinction is made between harvested and lying dead trees, which adds uncertainty to our analysis. Also, it would be worthwhile to give greater attention to documenting tree death in relation to disturbance events such as storms, insect calamities and large fires. Differences in census interval probably leads to some differences among regions in the distinction between harvest and mortality. The probability of a tree not yet having been salvaged is higher with a shorter census interval.

## Conclusions

For the first time ever, we have compiled a synchronized detailed data set of repeated tree measurements in NFIs from nine countries and 13 regions, from which actual harvest can be derived. These 714,000 re-measurements give for the first time a good insight in how different tree species were managed between 1990 and 2010 by various owners and in various regions in Europe.

From this large set of repeated tree measurements and the comparison with the growth and yield tables, we can conclude that there is no such thing as yield table harvesting in Europe. We found general trends of increasing harvest probability with higher productivity of the region and the species, but with important deviations related to local conditions like site accessibility, state of the forest resource (like age), specific subsidies, importance of other forest services, and ownership of the forest. As a result, we find a huge diversity in harvest regimes.

Over the time period covered in our inventories, the average harvest probability over all regions was 2.4% per year (in number of trees, DBH ≥ 120 mm) and the mortality probability was 0.4% per year. In Europe, harvest is thus by far the most important cause for trees to end their life. This confirms that Europe’s forests are regularly managed [64], at least in all our studied regions, and it is consistent with the low percentage of the forest that is reported to be unmanaged or reported as forest reserve [26, 73]. Our study not only confirms the importance of harvesting in Europe’s forest, but provides a clear approach for describing and quantifying its intensity. This approach can be used as an important cornerstone to document ‘sustainable forest management and intensity’ for the base period as required for the Forest Reference Level for EU Member States. For more information on defining ‘continuation of current forest management’ in order to set a Forest Reference Level based on the age class method we refer to [74]. Although in that EFISCEN based study rather standard management regimes were applied. Furthermore, the approach and the results can be used to improve harvest regimes in forest simulation models at various scales, allowing them to move from yield table regimes to actually observed harvesting patterns and intensities.

## Supporting information

S1 Fig Patterns of harvest and mortality probability over DBH for the four most important species per region. (DOCX)

S1 File Detailed results by region and tree species. (XLSX)

S2 File Patterns of harvest and mortality probability over DBH for all combinations of regions, species and owners. (XLSX)
