# Supplementary material for: Actual European forest management by region, tree species and owner based on 714,000 re-measured trees in national forest inventories
Source: PLoS One. 2018 Nov 12;13(11):e0207151. doi: 10.1371/journal.pone.0207151 (PMC6231657; doi:10.1371/journal.pone.0207151)
Supplement: S1 Fig — (DOCX) [file pone.0207151.s001.docx]

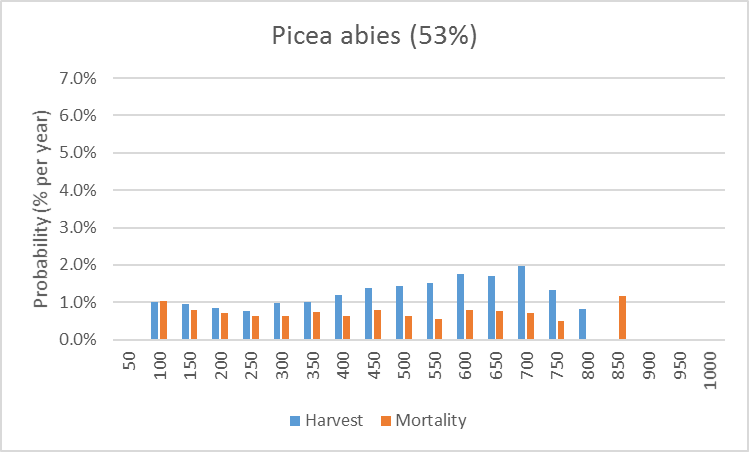

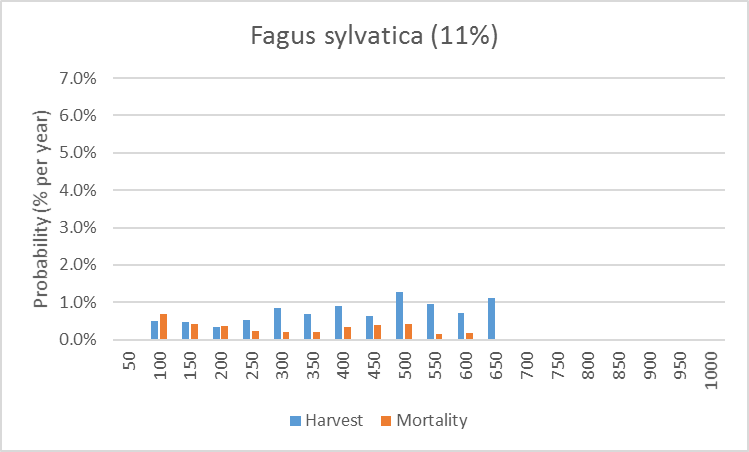

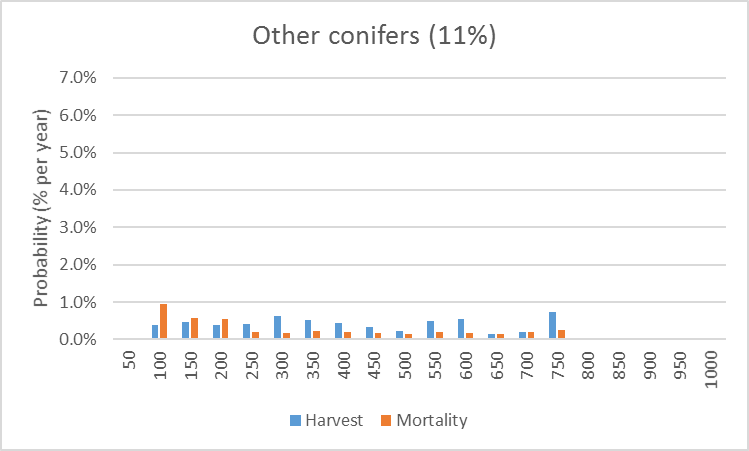

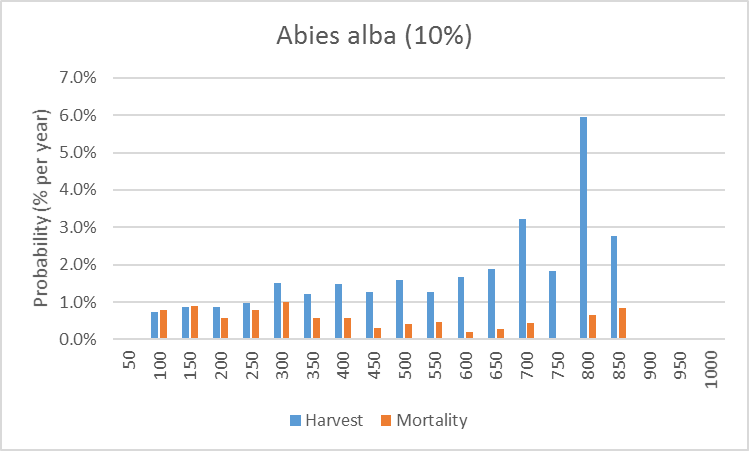


Figure A.1 Central Europe/ mountainous


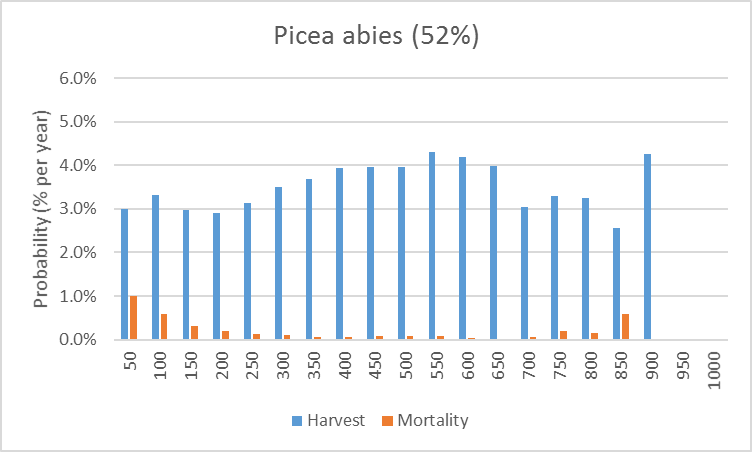

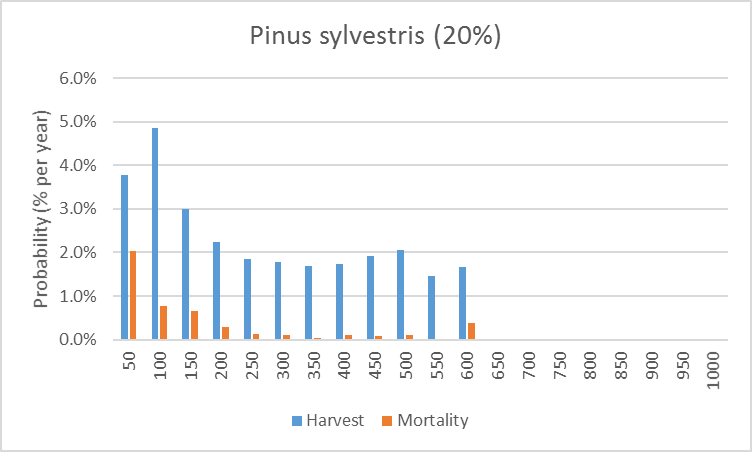

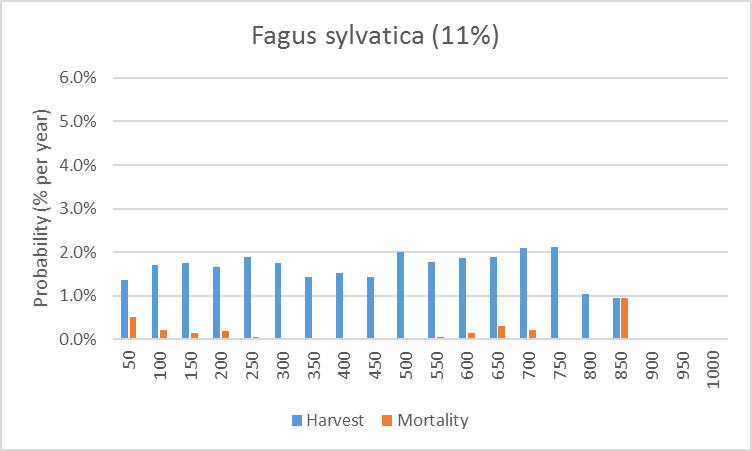

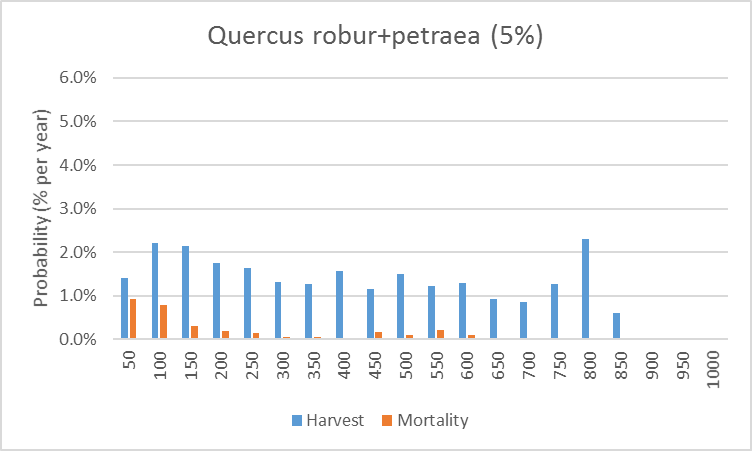


Figure A.2. Bavaria


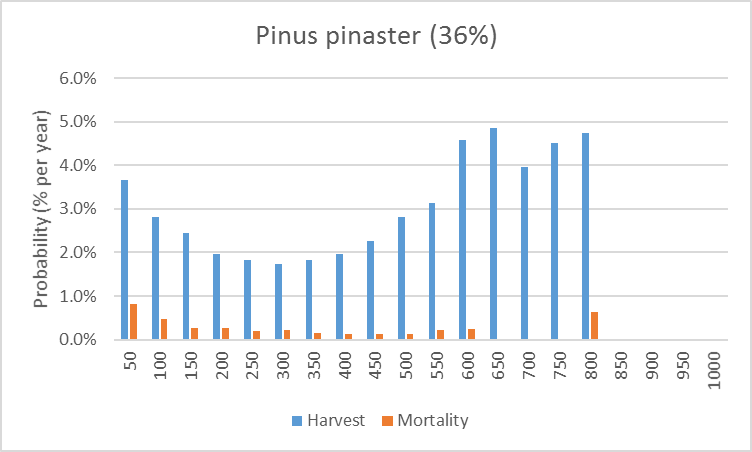

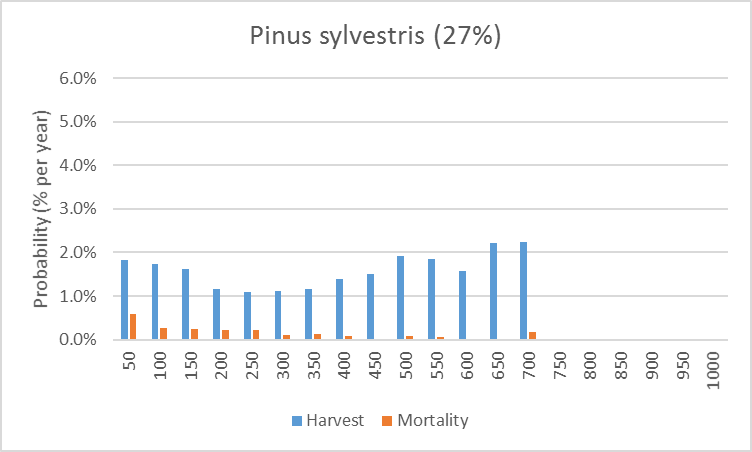

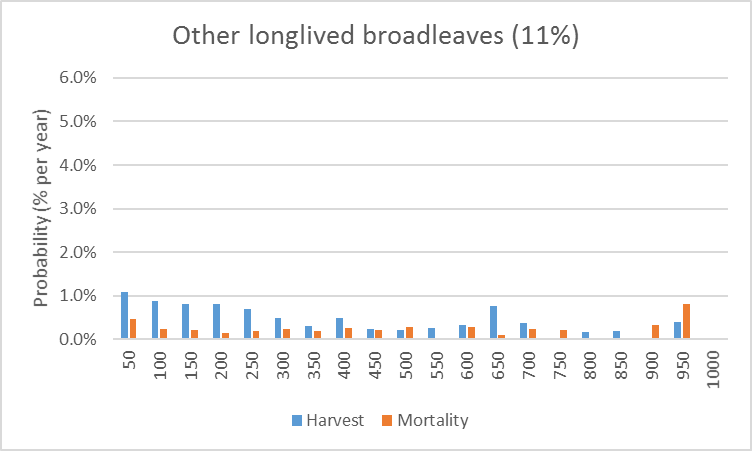

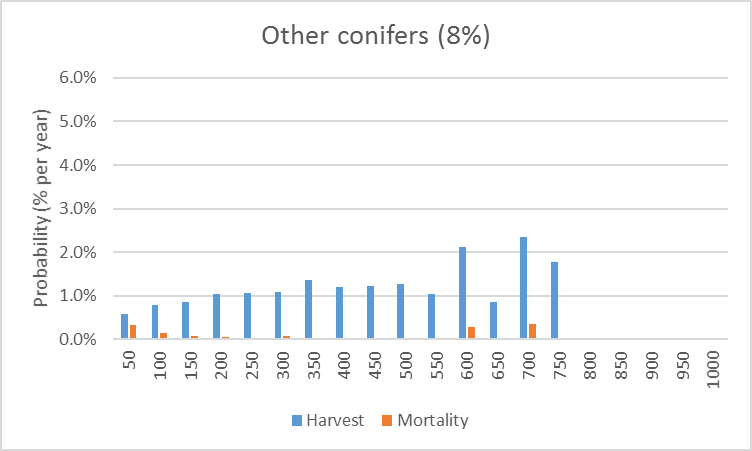


Figure A.3 Castilla y Léon


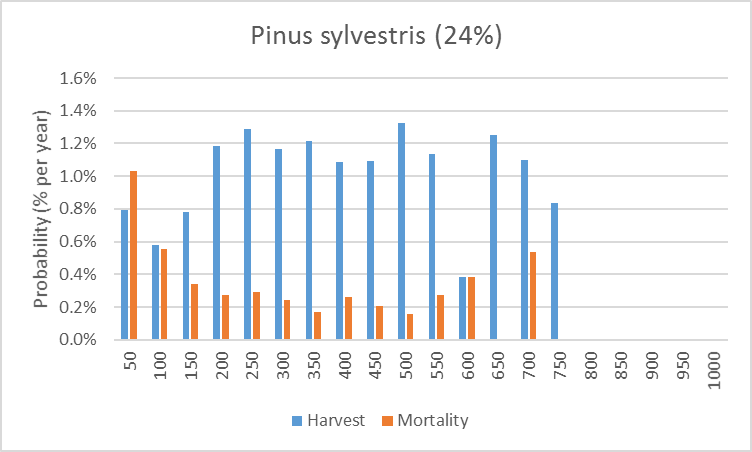

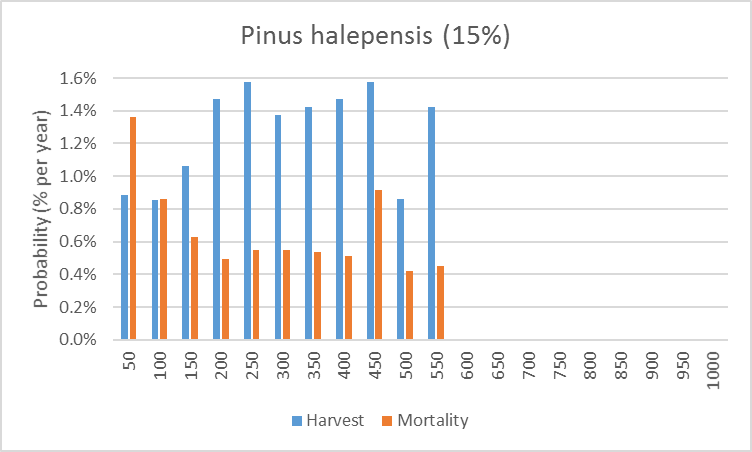

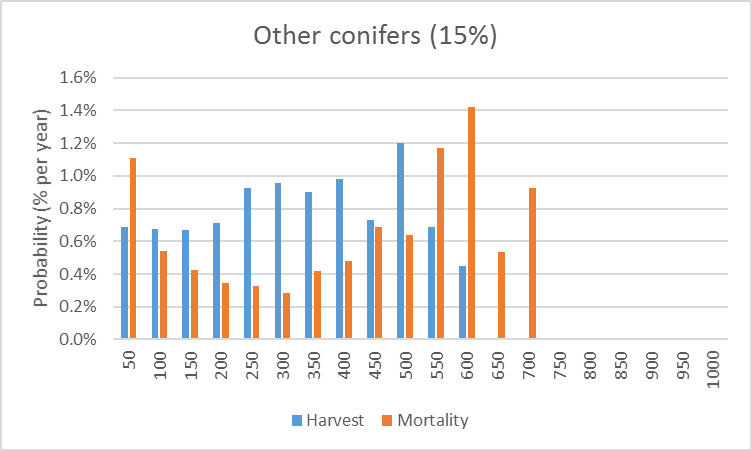

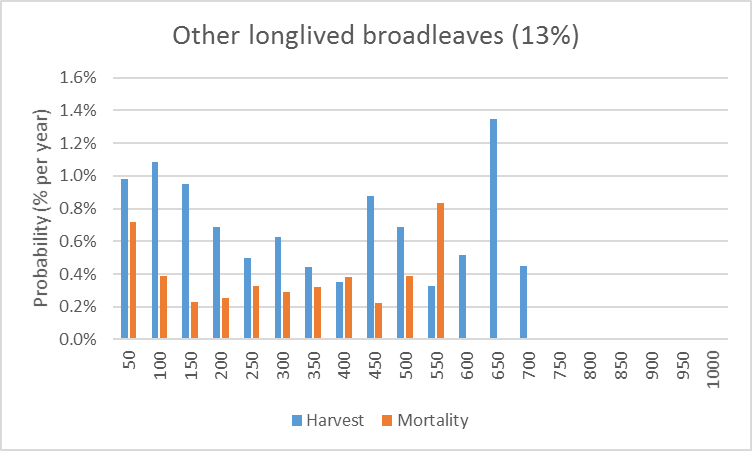


Figure A.4 Catalonia


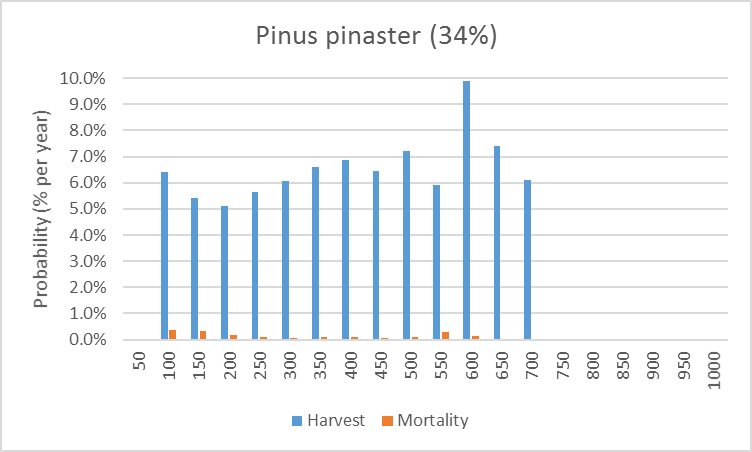

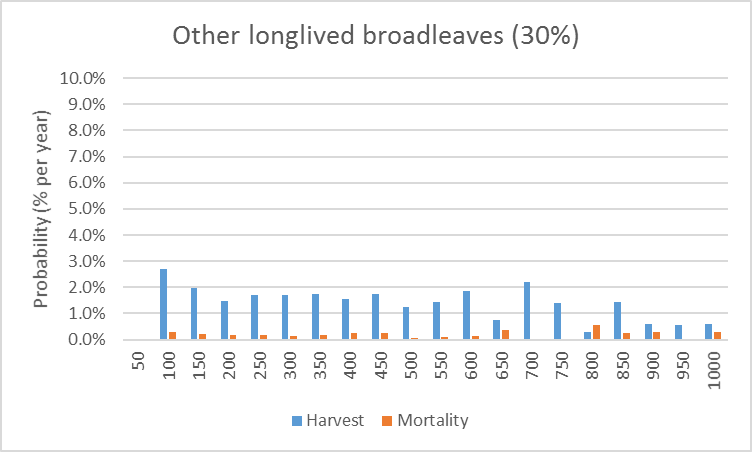

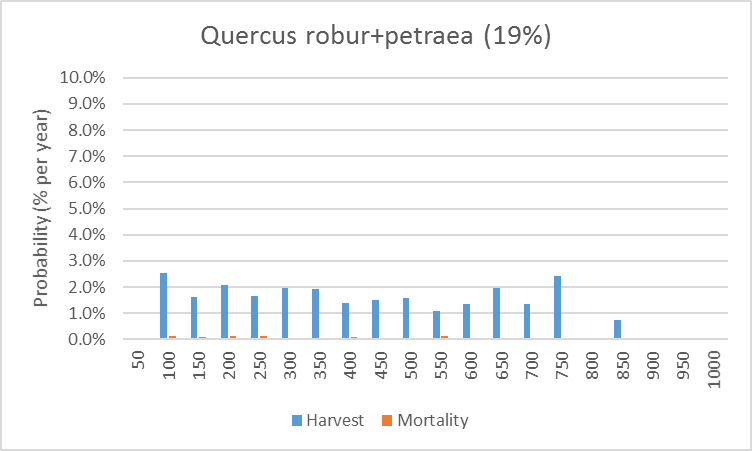

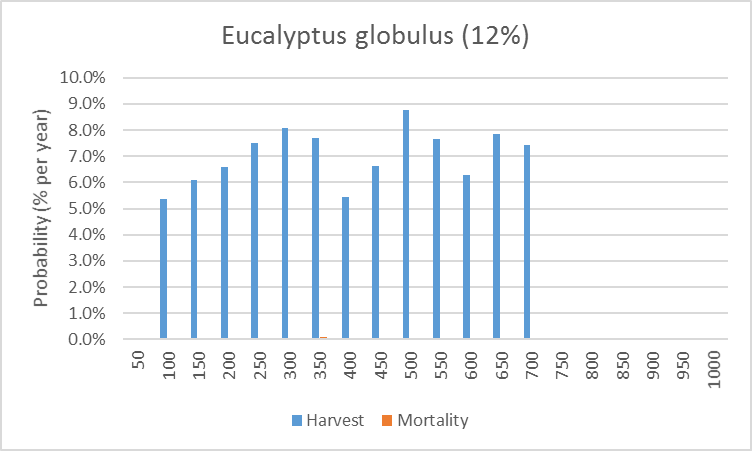


Figure A.5 Galicia


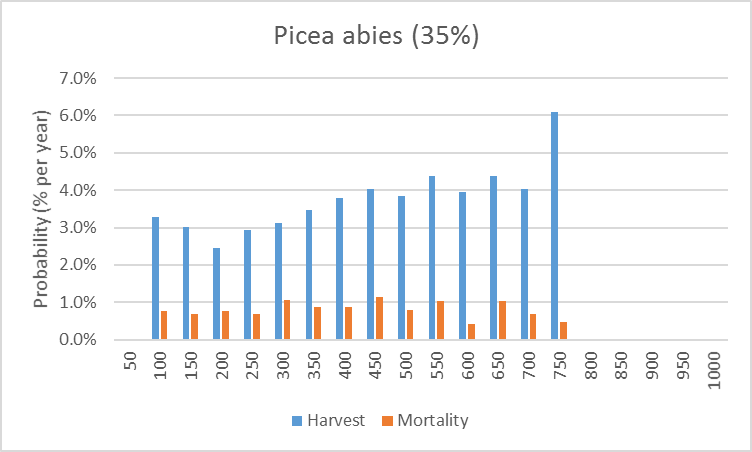

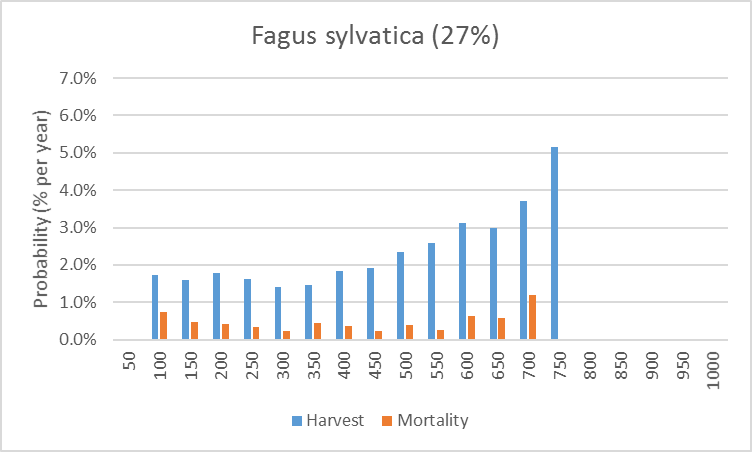

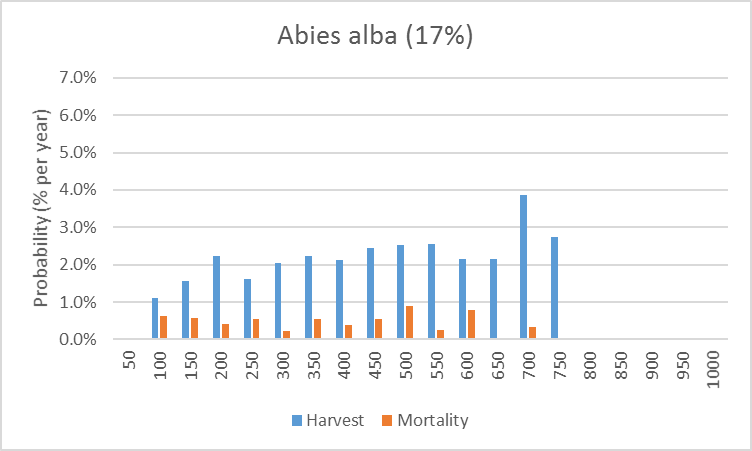

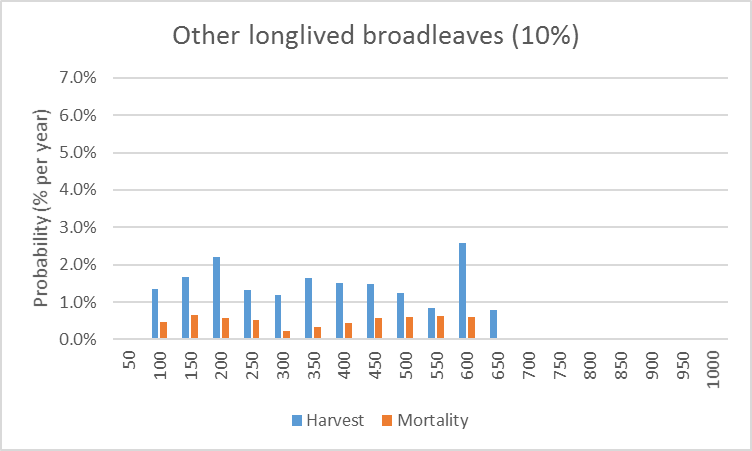


Figure A.6 Jura+Plateau


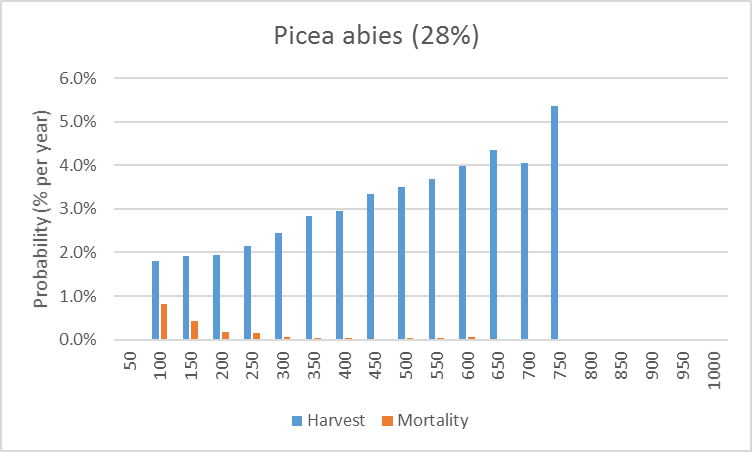

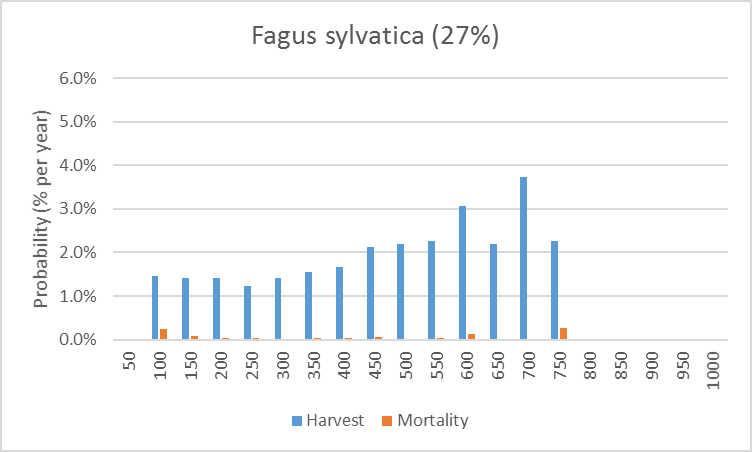


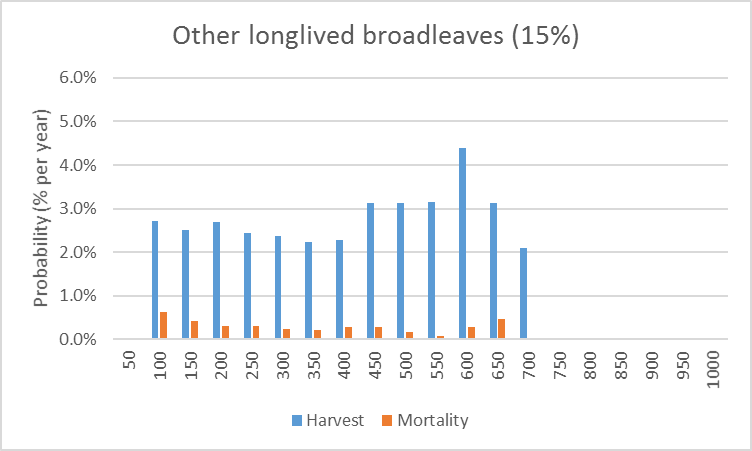

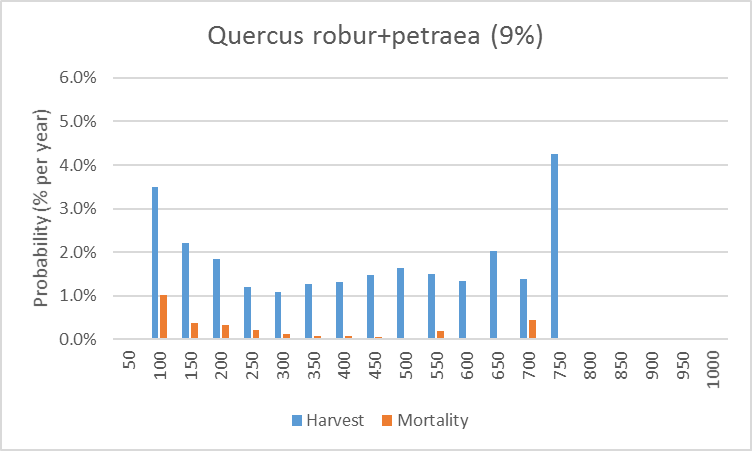


Figure A.7 Maribor


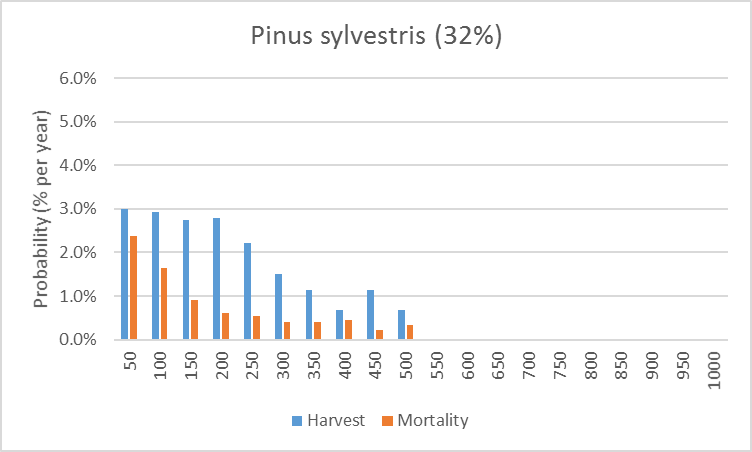

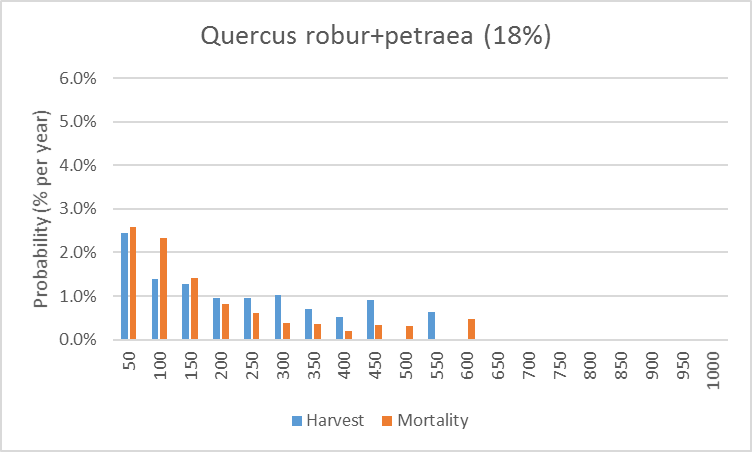

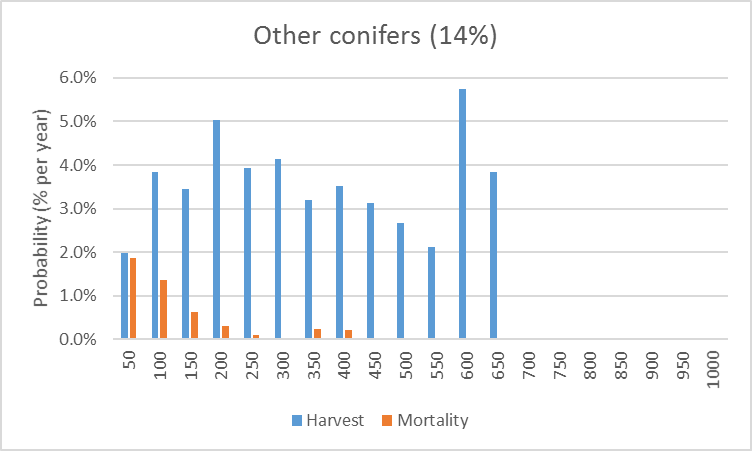

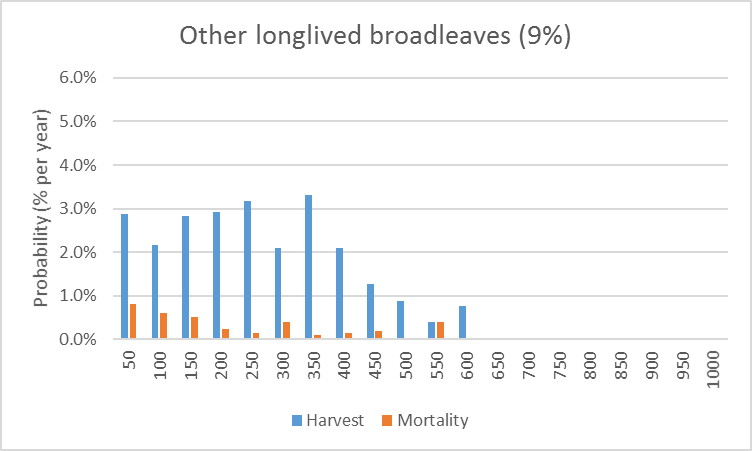


Figure A.8 The Netherlands


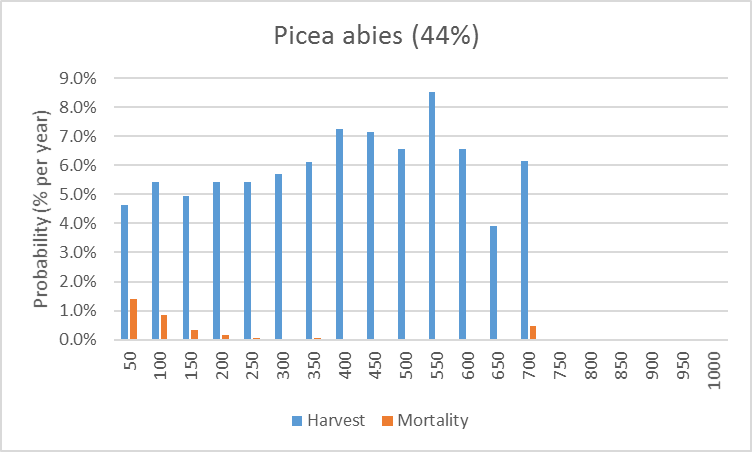

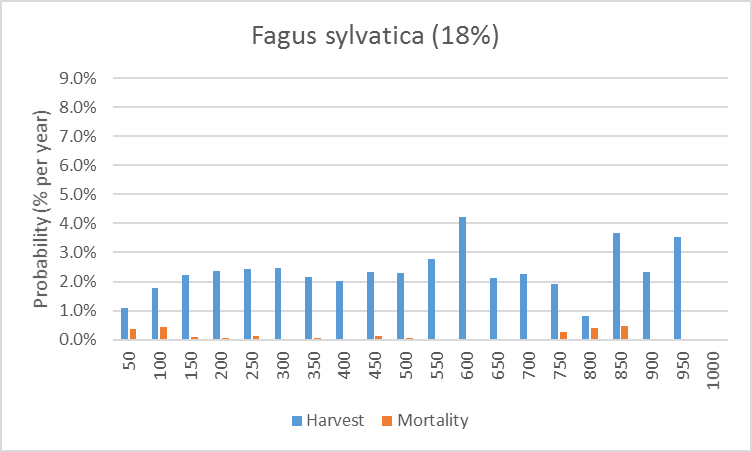

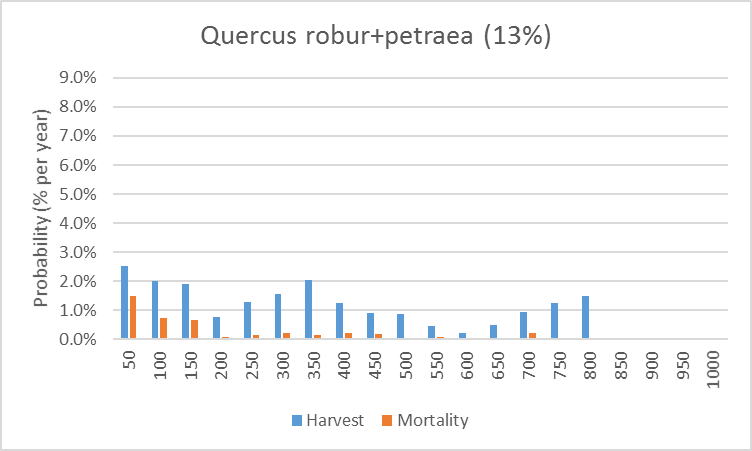

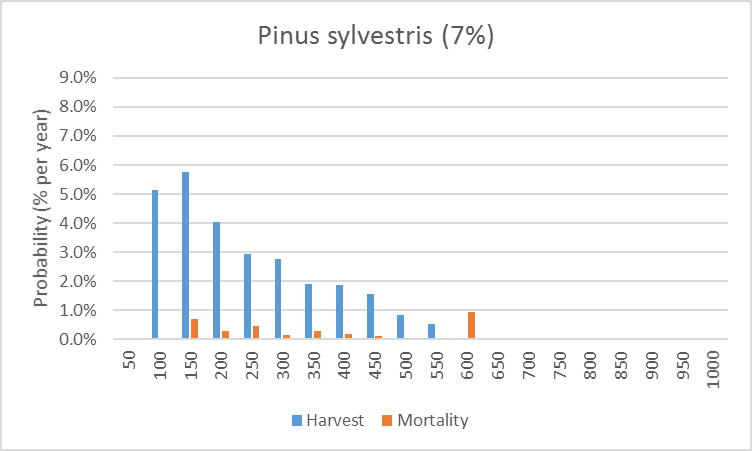


Figure A.9 Nordrhein-Westfalen


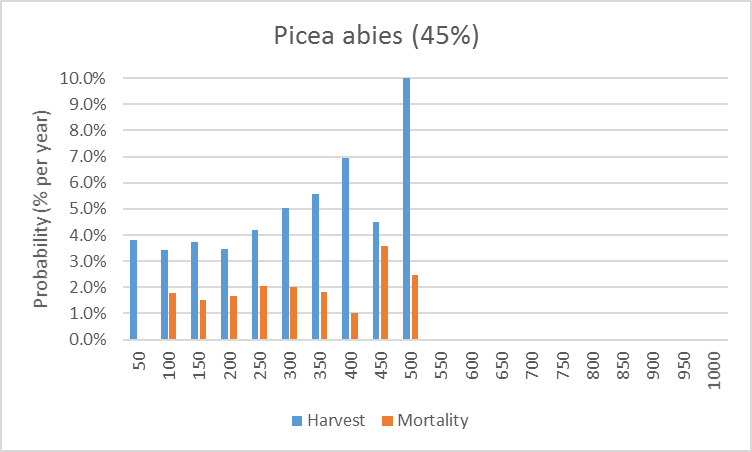

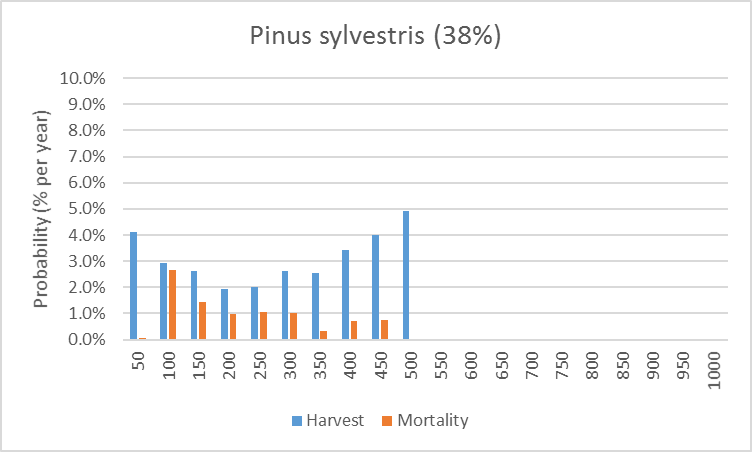

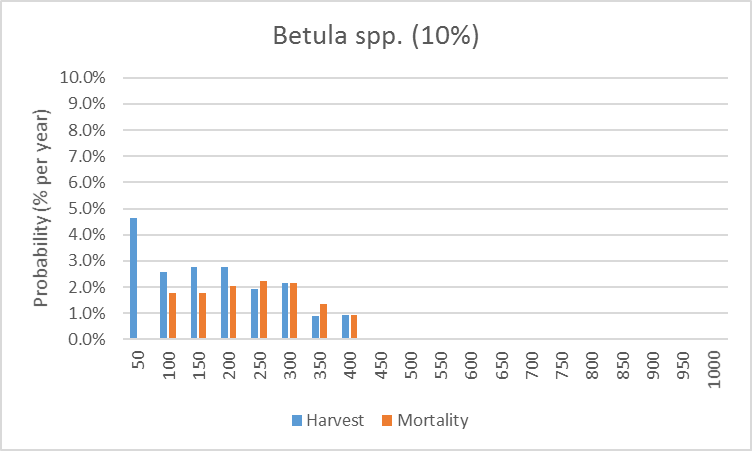

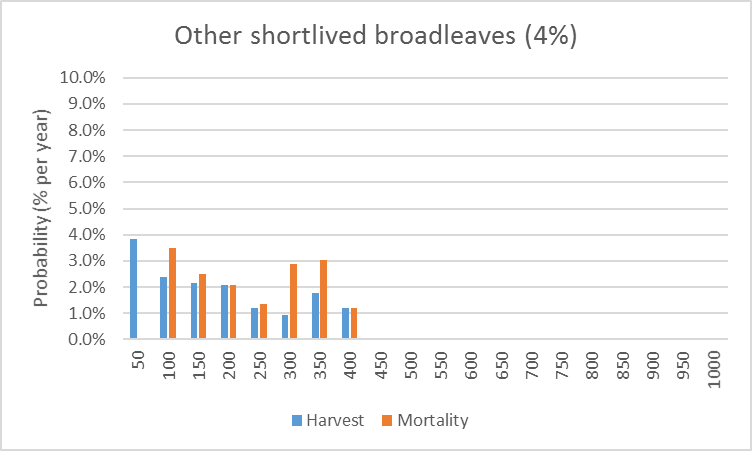


Figure A.10 Smaland


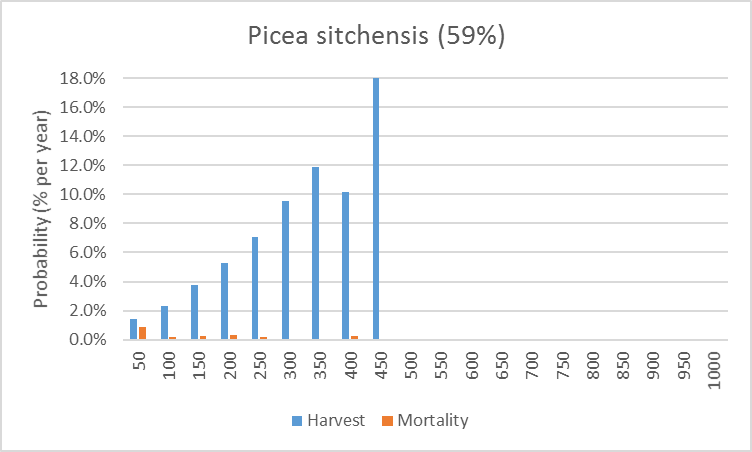

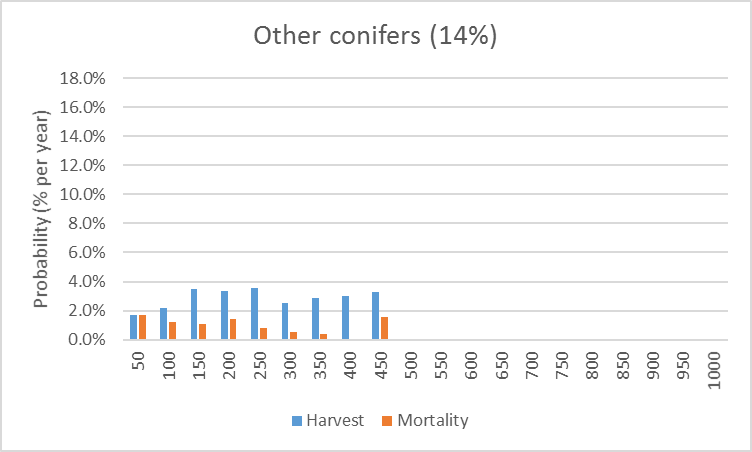

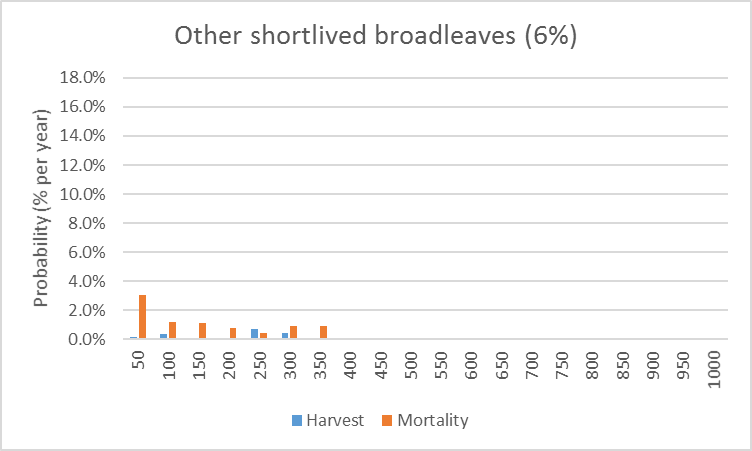

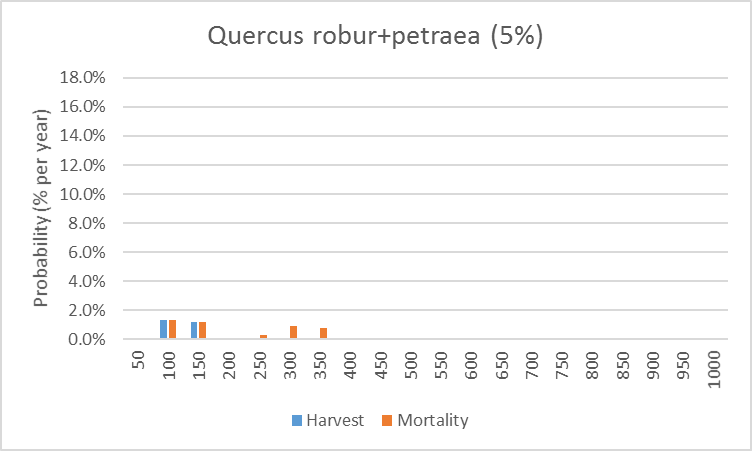


Figure A.11 Southwest Ireland


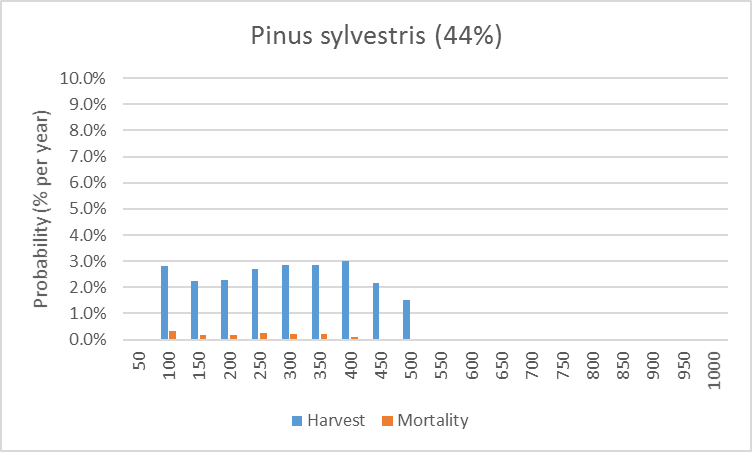

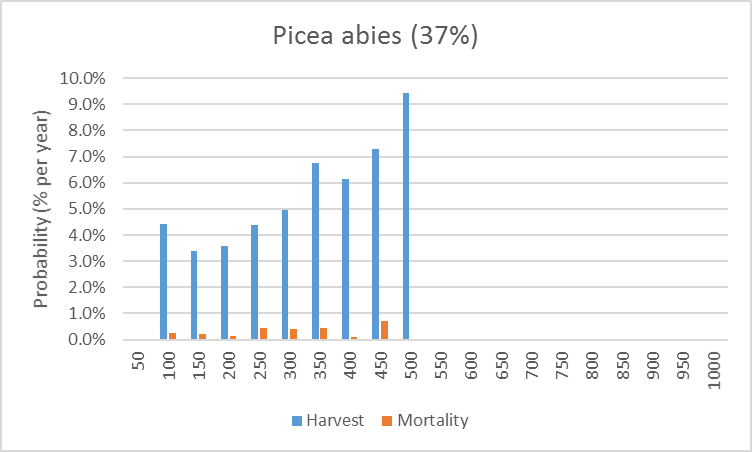

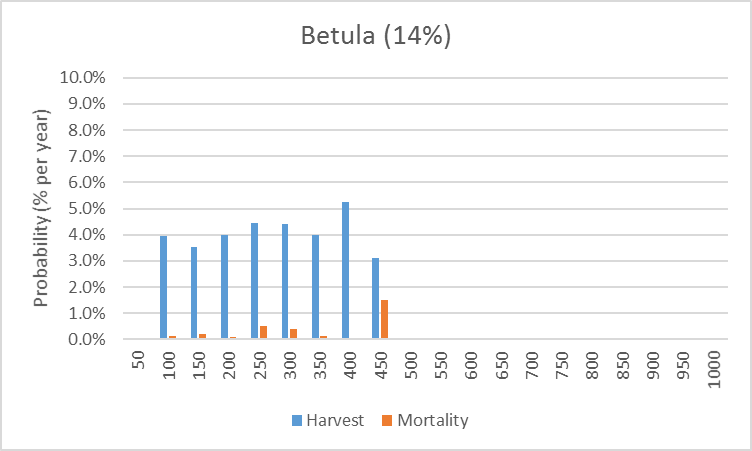

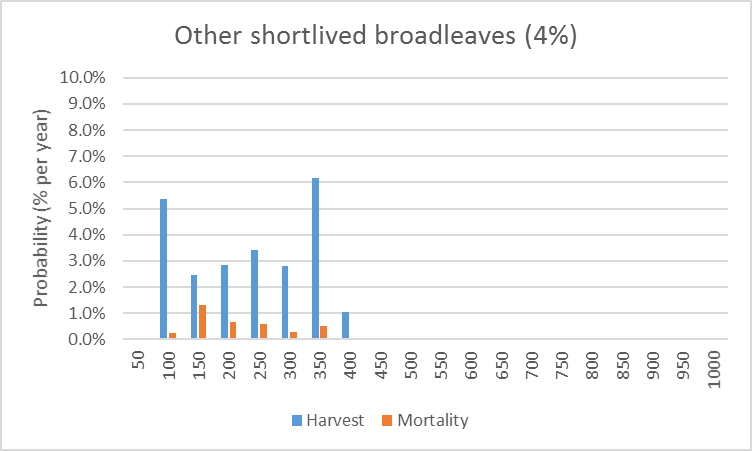


Figure A.12 South Finland


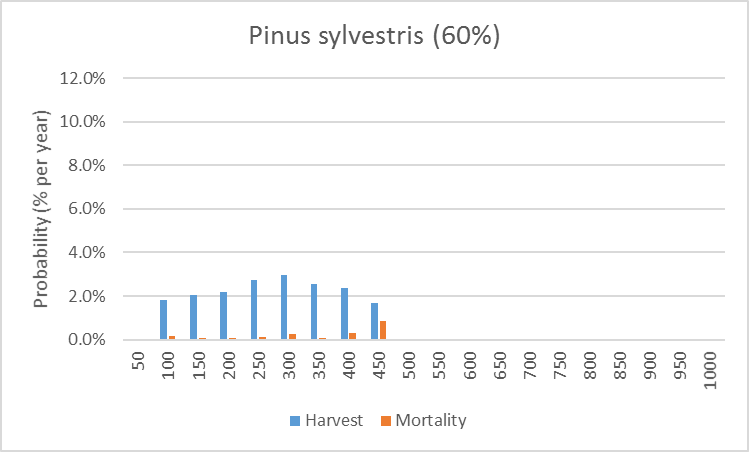

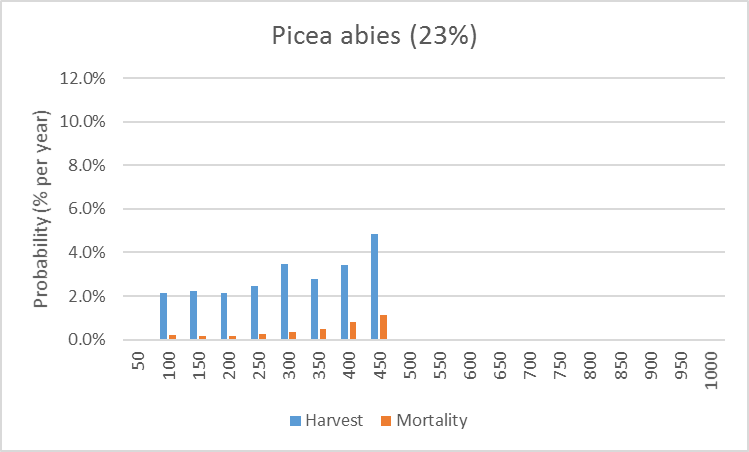

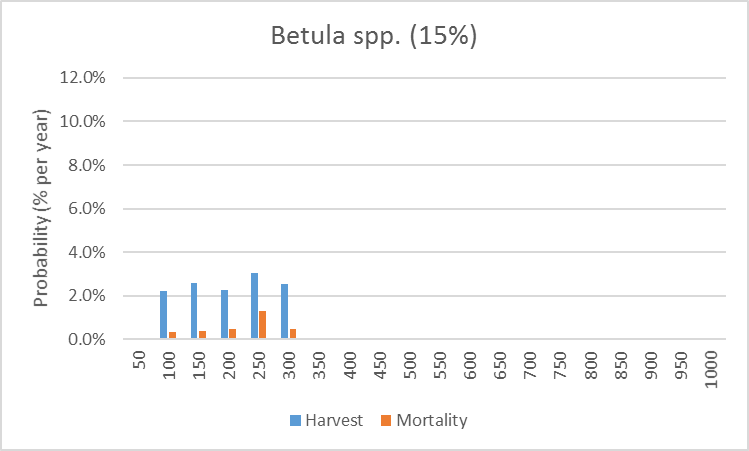

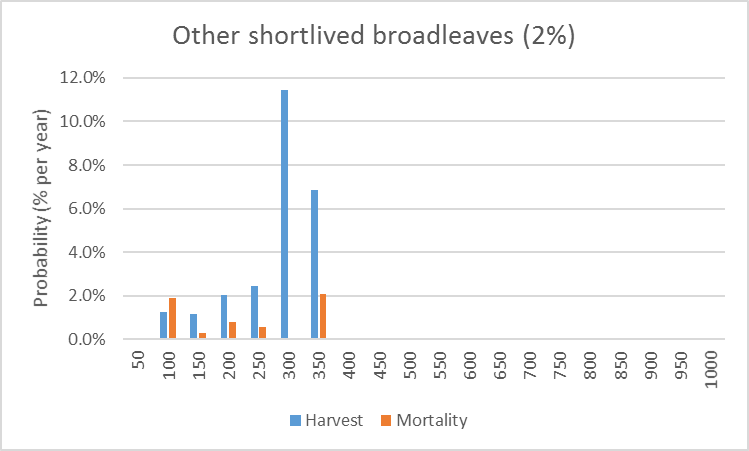


Figure A.13 North Finland
